# RNAi-dependent expression of sperm genes in ADL chemosensory neurons is required for olfactory responses in *Caenorhabditis elegans*

**DOI:** 10.3389/fmolb.2024.1396587

**Published:** 2024-07-11

**Authors:** Maria C. Ow, Mailyn A. Nishiguchi, Abdul Rouf Dar, Rebecca A. Butcher, Sarah E. Hall

**Affiliations:** ^1^ Biology Department, Syracuse University, Syracuse, NY, United States; ^2^ Department of Chemistry, University of Florida, Gainesville, FL, United States

**Keywords:** *C. elegans*, dauer, postdauer, ADL, NRDE-3, major sperm protein, *gsp-3*

## Abstract

Environmental conditions experienced early in the life of an animal can result in gene expression changes later in its life history. We have previously shown that *C. elegans* animals that experienced the developmentally arrested and stress resistant dauer stage (postdauers) retain a cellular memory of early-life stress that manifests during adulthood as genome-wide changes in gene expression, chromatin states, and altered life history traits. One consequence of developmental reprogramming in *C. elegans* postdauer adults is the downregulation of *osm-9* TRPV channel gene expression in the ADL chemosensory neurons resulting in reduced avoidance to a pheromone component, ascr#3. This altered response to ascr#3 requires the principal effector of the somatic nuclear RNAi pathway, the Argonaute (AGO) NRDE-3. To investigate the role of the somatic nuclear RNAi pathway in regulating the developmental reprogramming of ADL due to early-life stress, we profiled the mRNA transcriptome of control and postdauer ADL in wild-type and *nrde-3* mutant adults. We found 711 differentially expressed (DE) genes between control and postdauer ADL neurons, 90% of which are dependent upon NRDE-3. Additionally, we identified a conserved sequence that is enriched in the upstream regulatory sequences of the NRDE-3-dependent differentially expressed genes. Surprisingly, 214 of the ADL DE genes are considered “germline-expressed”, including 21 genes encoding the Major Sperm Proteins and two genes encoding the sperm-specific PP1 phosphatases, GSP-3 and GSP-4. Loss of function mutations in *gsp-3* resulted in both aberrant avoidance and attraction behaviors. We also show that an AGO pseudogene, Y49F6A.1 (*wago-11*), is expressed in ADL and is required for ascr#3 avoidance. Overall, our results suggest that small RNAs and reproductive genes program the ADL mRNA transcriptome during their developmental history and highlight a nexus between neuronal and reproductive networks in calibrating animal neuroplasticity.

## 1 Introduction

Exposure to stressful environmental conditions early in the life of an animal can result in changes of gene expression that can be manifested in adulthood (Fusco and Minelli, 2010; Xue and Leibler, 2018; Rodrigues and Beldade, 2020; Sommer, 2020). The nematode *Caenorhabditis elegans* responds to early-life stress conditions, such as starvation, high temperature, or crowding, through entry into an alternative life history trajectory (Hu, 2018). Upon exposure to these unfavorable growth conditions, L2d larvae enter a stress resistant, developmentally arrested, and non-feeding stage called dauer, at which they can remain for months. Dauer larvae exit as postdauers (PD) L4 larvae only when environmental conditions improve (Cassada and Russell, 1975). Animals that experience favorable conditions in early life continuously develop through four larval stages (L1-L4) before reaching reproductive adulthood (control adults, CON) (Sulston and Horvitz, 1977). We have previously shown that PD adults retain a cellular memory of passage through dauer that manifests as altered genome-wide gene expression, small RNA populations, chromatin states, and life history traits compared to control adult animals (Hall et al., 2010; 2013; Ow et al., 2018; Ow et al., 2021).

One target of this developmental programming is the *osm-9* TRPV channel gene whose expression is downregulated in the ADL chemosensory neurons of PD adults (Sims et al., 2016). TRPV channels are members of a superfamily of evolutionarily conserved transient receptor potential (TRP) cation channels found in all eukaryotes and form homo- or heterocomplexes with other TRPs to regulate sensory perception (Kahn-Kirby and Bargmann, 2006; Nilius and Owsianik, 2011). In *C. elegans*, mutants of TRPV (vanilloid family) channel genes, such as those encoding for OSM-9 and its heteromeric TRPV channel partner member, OCR-2, exhibit defects in olfaction, nociception, and animal behavior. The *osm-9* gene is expressed in the sensory cilia and in several sensory neurons including ADL (Colbert et al., 1997; Tobin et al., 2002; Kahn-Kirby and Bargmann, 2006; Jang et al., 2012). The reduced *osm-9* mRNA expression in postdauer ADL neurons results in the abrogation of avoidance to ascr#3 (asc-ΔC9), which is an *osm-9*-dependent, ADL-mediated behavior (Sims et al., 2016). Ascr#3 is a dauer-inducing small signaling molecule with a nine-carbon α,β-unsaturated carboxylic acid side chain that is a component of pheromones produced by *C. elegans* that are crucial for olfactory behavior, aggregation, and mating (Butcher et al., 2007; Ludewig and Schroeder, 2018). Our early work indicated that TGF-ß signaling, chromatin remodeling, and endogenous small RNA interference (RNAi) pathways contribute to the mechanism by which *osm-9* expression is reprogrammed in PD adults due to early-life environmental stress (Sims et al., 2016).

Endogenous small RNA pathways in animals are categorized into three major classes based on their biogenesis and their effector protein partners: PIWI-interacting RNA (piRNAs), microRNAs (miRNAs), and endogenous small interfering RNAs (endo-siRNAs). These small non-coding RNAs (snRNAs) range between ∼20 and 30 nucleotides (nt) long and are fully or partially anti-sense to their target transcripts (Billi et al., 2014; Ambros and Ruvkun, 2018; Bartel, 2018; Czech et al., 2018; Ketting and Cochella, 2021). In *C. elegans*, the biogenesis of endo-siRNAs starts with double-stranded RNA (dsRNA) being processed by a protein complex that includes the RNase III endoribonuclease DICER into two classes of 26 nt long primary siRNAs that have a 5′ guanosine (26Gs): ERGO-1 26G RNAs and 26G RNAs that associate with two paralogous Argonautes (AGOs), ALG-3 and ALG-4 (ALG-3/4) (Billi et al., 2014; Ketting and Cochella, 2021). The ERGO-1 26G RNAs primarily target transcripts produced in the oogenic germline, embryos, pseudogenes, and gene duplications while ALG-3/4 26G RNAs are derived from the spermatogenic germline (Han et al., 2009; Conine et al., 2010; Gent et al., 2010; Vasale et al., 2010). 26G RNAs stimulate the amplification of 22 nt long secondary siRNAs with a 5’ guanosine (22Gs) that are further subdivided into two categories, those that bind to the Worm specific AGO (WAGO) clade and those that bind to the CSR-1 AGO (Guang et al., 2008; Claycomb et al., 2009; Gu et al., 2009; Burton et al., 2011; Ashe et al., 2012; Buckley et al., 2012; Luteijn et al., 2012; Shirayama et al., 2012). The WAGO-bound 22G RNAs effect the silencing of target transcripts while CSR-1-bound 22G RNAs do not silence their targets but rather promote the expression of germline genes and proper chromosome segregation (Yigit et al., 2006; Claycomb et al., 2009).

Out of 26 AGO proteins encoded in *C. elegans*, 19 have been attributed as functional, most of which remain uncharacterized or partially characterized (Ketting and Cochella, 2021; Seroussi et al., 2023). While most characterized AGOs function primarily in the germ line, only four AGOs are reported to be expressed in neurons: ERGO-1, NRDE-3, and the miRNA-specific ALG-1 and ALG-2 (Seroussi et al., 2023). We previously showed that NRDE-3 and its nuclear complex members are required for the downregulation of *osm-9* expression in PD ADL neurons and the resulting abrogation of the avoidance response to ascr#3 (Sims et al., 2016). NRDE (nuclear RNAi defective)-3/WAGO-12 is the effector protein of the somatic nuclear RNAi pathway and associates with 22G RNAs (Guang et al., 2008; 2010; Gent et al., 2010; Burton et al., 2011). Once bound to 22G RNAs, cytoplasmic NRDE-3 translocates to the nucleus where it is directed to target nascent pre-mRNA and recruits NRDE-2 to inhibit RNA polymerase II transcription elongation (Guang et al., 2008). NRDE-3 and NRDE-2 then recruit NRDE-1 to the nascent pre-mRNA to potentiate its association with chromatin and the trimethylation of Histone 3 Lysine 9 (H3K9me3) at the targeted locus (Guang et al., 2010; Burkhart et al., 2011). The small RNA-directed chromatin modification, but not the association with pre-mRNA, by NRDE-1 depends on a fourth nuclear RNA pathway factor, NRDE-4 (Guang et al., 2008; 2010; Burkhart et al., 2011). Similarly, NRDE-1, NRDE-2, and NRDE-4 also participate in the germline nuclear RNAi pathway and transgenerational epigenetic inheritance (TEI) directed by the AGO HRDE-1/WAGO-9 (Ashe et al., 2012; Buckely et al., 2012; Luteijn et al., 2012).

Compared to the prominence of endo-siRNAs in germline function, maintenance, and integrity, our knowledge of their role in the *C. elegans* nervous system is limited (Ow and Hall, 2021). Several reports have emerged in recent years describing their critical neural function. Mutations in genes encoding for CSR-1 and a component of the *Mutator* foci, a perinuclear structure where production of siRNA occurs, MUT-16 (Phillips et al., 2012), disrupt dauer formation due the exposure to starvation, crowding, and high temperature (Bharadwaj and Hall, 2017). This dauer defective (*daf-d*) phenotype was rescued by the expression of *mut-16* using a pan-neuronal promoter. In addition, MUT-16 and CSR-1 are required for the expression of the *gpa-1*, *gpa-3* and *gpc-1* G proteins whose mutations are associated with the *daf-d* phenotype, suggesting that endogenous RNAi pathways play a role in the phenotypic plasticity of an animal in response to the environment (Bharadwaj and Hall, 2017). *C. elegans*’ attraction towards the odorant benzaldehyde was found to be heritable for at least two generations in the absence of the original odorant trigger through neuronal endo-siRNAs transmitted through the germ line. Animals expressing a co-factor necessary for the biogenesis of 26G RNAs, RDE-4 (Duchaine et al., 2006; Lee et al., 2006; Vasale et al., 2010), from a neuronal-specific promoter, led to the identification of neuronal small RNAs that affected the expression of germline mRNAs. Some of these germline gene expression changes perdured for at least three generations and were dependent on the germline-specific nuclear RNAi pathway AGO HRDE-1 silencing the *saeg-2* gene encoding a component of the SAEG-1/SAEG-2 histone deacetylase complex in response to benzaldehyde (Posner et al., 2019). Loss of NRDE-3 results in defective olfactory adaptation behavior to the odorant butanone mediated by the AWC olfactory sensory neurons. Accordingly, an increase in 22G RNAs antisense to the *odr-1* guanylyl cyclase gene required for AWC function was found, suggesting that a NRDE-3-mediated reduction in *odr-1* expression in AWC is responsible for *C. elegans’* olfactory adaptation to butanone (Juang et al., 2013).

While much is known about NRDE-3 in regulating somatic nuclear small RNA pathway triggered by exogenous substrates (Guang et al., 2008; 2010; Gent et al., 2010; Burkhart et al., 2011; Burton et al., 2011), its role in endogenous gene expression is yet to be well-characterized. To further investigate the role of NRDE-3 in regulating the developmental reprogramming of gene expression in ADL neurons due to early-life crowding stress, we profiled the mRNA transcriptome of control and postdauer ADL in wild-type and *nrde-3* mutant adults. We found that hundreds of genes typically associated with the germ line, such as those encoding for *major sperm protein* (MSP) gene family, are also differentially expressed in ADL neurons in a NRDE-3 dependent manner. Mutation in the PP1 phosphatase, GSP-3, which is required for MSP function in sperm, significantly decreases avoidance behavior in response to nociceptive stimuli and the chemotaxis behavior towards attractive stimuli. We demonstrate that germline RNAi AGO, WAGO-4, and miRNA AGO, ALG-1, are expressed in ADL and modulate ascr#3 avoidance. Furthermore, we show that AGO WAGO-11, previously classified as a pseudogene, is expressed in ADL and is required for its function. Together, our results highlight a nexus between neuronal and reproductive networks in recalibrating the life history and the neuroplasticity of an animal in response to early-life environmental experience.

## 2 Materials and methods

### 2.1 *C. elegans* strains and husbandry

Worms were grown at 20°C in Nematode Growth Medium (NGM) seeded with *E. coli* OP50 (Stiernagle, 2006) unless otherwise stated. Cultivation of worms used for FACS were grown on NGM plates (100 mm petri dishes) seeded with 20× concentrated *E. coli* OP50 at 20°C. PD_Phe_ adults were obtained as previously reported (Ow et al., 2018). All worm strains used in this study are listed in Supplementary Table S1. All un-backcrossed mutant strains were backcrossed 4–6 times into our laboratory N2 Bristol wild-type strain before use.

### 2.2 Transgenic strains

SEH306 *pdrEx80 (sre-1*p*::gfp; unc-122*p*::dsRed*) was made by injecting N2 adults with a plasmid containing 3 kb of the *sre-1* promoter fused upstream of *gfp* (*sre-1*p*::gfp*) and a *unc-122*p*::dsRed* reporter as the co-injection marker. The *sre-1*p*::gfp; unc-122*p*::dsRed* extrachromosomal array was integrated using a UV crosslinker and backcrossed 6x with our N2 laboratory strain to create SEH318 *pdrIs9* (*sre-1*p*::gfp; unc-122*p*::dsRed*). SEH318 was crossed into YY158 *nrde-3(gg66) X* to build SEH321 *nrde-3(gg66) X; pdrIs9* (*sre-1*p*::gfp; unc-122*p*::dsRed)*. All mutant strains expressing *pdrIs9 (sre-1*p*::gfp; unc-122*p*::dsRed)* were built by genetically crossing SEH318 into each mutant strain and confirmed by PCR or Sanger sequencing (Supplementary Table S1).

### 2.3 Adult neuronal isolation

Isolation of adult ADL neurons of SEH318 *pdrIs*9 (*sre-1*p*::gfp; unc-122*p*::dsRed*) CON and PD_Phe_ adults and SEH321 *nrde-3(gg66) X*; *pdrIs9* (*sre-1*p*::gfp; unc-122*p*::dsRed*) CON and PD_Phe_ adults were done as described by (Kaletsky et al., 2016). Briefly, staged 1-day old adult animals grown at 20°C on 100 mm NGM plates seeded with 20x concentrated OP50 were harvested and washed with M9 buffer until the supernatant was clear. 750 μL of Neuronal Isolation Lysis Buffer (200 mM DTT; 0.25% SDS; 20 mM HEPES pH 8.0; 3% sucrose) was added to a packed pellet of worms (up to 250 μL) and rotated at room temperature for 6.5 min. The worm mixture was then washed with M9 and 500 μL of a freshly made pronase (Sigma P6911) solution (20 mg/mL) was added and vigorous pipetted for 100 times (∼40 s) in 2 min intervals until ADL head neurons dissociated from the worm body as monitored using fluorescence microscopy. Once worm heads disintegrated (∼14–16 min), the pronase reaction was stopped with 250 μL of 1x PBS buffer supplemented with 2% Fetal Bovine Serum (Fisher Scientific 10082139) and iced. The worm mixture was then filtered through 5 μM filter, stored on ice, and immediately transported to the cell sorting facility (∼15 min interval) where it was sorted upon arrival.

### 2.4 Fluorescence activated cell sorting (FACS)

ADL chemosensory neurons were collected from a neuronal population obtained from 1-day old adults using with a Becton Dickinson FACS Aria III Cell Sorter. Neurons collected from 1-day old wild-type N2 adults were used to set the FACS gate. The average total FACS event for a strain and condition ranged between 128,229 and 846,079. FACS events were collected directly into TRIzol (Life Technologies) and immediately frozen in dry ice. All frozen samples were stored at −80°C until ready for processing.

### 2.5 RNA extraction, mRNA-Seq library preparation, and data analyses

Total RNA extraction of FACS events was done using NEB Monarch Total RNA Miniprep (New England Biolabs [NEB] T2010). Four biological independent RNA-Seq libraries for a strain and a condition were prepared using NEBNext^®^ Poly(A) mRNA Magnetic Isolation Module (NEB E7490S) and NEBNext^®^ Ultra™ II RNA Library Prep with Sample Purification Beads (NEB E7775S) following the recommendations of the manufacturer. Indexing of libraries was done using NEBNext^®^ Multiplex Oligos for Illumina^®^ (Index Primers Set 1) (NEB E7335S) and NEBNext^®^ Multiplex Oligos for Illumina^®^ (Index Primers Set 2) (NEB E7500S) according to the manufacturer’s instructions. Data analysis was performed on the CLC Genomics Workbench (Qiagen) with differential expression assessed using EdgeR (Robinson, McCarthy, and Smyth, 2010) as previously described (Ow et al., 2018). Raw data can be accessed through NCBI GEO GSE268801.

### 2.6 Gene ontology (GO) term analyses

GO terms enrichment was determined using Gorilla (Eden et al., 2009).

### 2.7 Statistical significance

Statistical significance was determined using GraphPad Prism version 9.5 (San Diego, CA, United States). The significance of hypergeometric distributions was determined using a hypergeometric *p*-value software calculator (https://systems.crump.ucla.edu/hypergeometric/index.php).

### 2.8 Bioinformatics analysis of the PD motif

To identify genes whose upstream regulatory sequence contain the DAF-3/SMAD binding site and the conserved sequence of the PD motif, a custom BLAST database including 500 bp upstream of ATG translational start sites for all genes in the *C. elegans* genome was created using NCBI. This database was used to search for genes whose 5′UTR contained “GTCTA” or the “CT[AT]TAA[AT]TTN(0,3)[AC]AN(0,2)TTTTG[CT]CATAA[TA]C[TC]” pattern, where “N” could be any nucleotide and the numbers signify the range of possible number of nucleotides. The overlap of the genes in each list were genes that contained the full PD motif. The python script used for this analysis can be found at https://github.com/mnishiguchi7/Hall_Lab/blob/main/script.

### 2.9 Single molecule fluorescence *in situ* hybridization (smFISH)

One-day old adults expressing the *sre-1*p*::gfp* transgene were used for smFISH (Ji and Oudenaarden, 2012) and carried out as previously described (Sims et al., 2016). Custom Stellaris™ smFISH probe sets consisting of 27 probes for *msp-19* (Quasar^®^ 570) and 32 probes for *gfp* (Quasar^®^ 670) were designed using the Stellaris™ probe designer website (LGC Biosearch Technologies). Probe sequences will be made available upon request. Images were acquired on a Leica DM5500 B microscope equipped with a Leica CTR5500 electronic box with a Hamamatsu Digital Camera C10600 ORCA R2 using maximally projected images of z-stacks as optimized by the Leica LAS AF 3.1.0 software followed by quantification on ImageJ. SmFISH experiments were done using two independent biological samples.

### 2.10 Immunofluorescence

One-day old adults expressing the *sre-1*p*::gfp* transgene were used for immunofluorescence using the method reported by Serrano-Saiz et al. (2017). In brief, 1-day old adults were fixed overnight with 1x PBS supplemented with 4% paraformaldehyde (Thermo Fisher AA433689M) followed by washing in PT Buffer (1x PBS, 0.5% Triton X-100). Fixed worms were reduced for ∼18–20 h at 37°C with 5% ß-mercaptoethanol in 1% Triton X-100 and 0.1 M Tris-HCl pH 7.5 followed by washing with TT Buffer (1% Triton X-100, 0.1 M Tris-HCl pH 7.5) and CTT Buffer (1 mM CaCl_2_, 1% Triton X-100, 0.1 M Tris-HCl pH 7.5). Worms were pelleted and shaken at room temperature for ∼30 min in CTT Buffer supplemented with 1 mg/ml of collagenase Type IV (Millipore Sigma C5138) until they fragmented (20%–50%). Fragmented worm samples were rinsed with PT Buffer and incubated with 1x PBS supplemented with 1 mg/mL of NaBH4 (Millipore Sigma 71320) for 1 h at 4°C with rotation. Samples were washed with PT Buffer and blocked in 0.2% gelatin solution [1x PBS, 0.5% Triton X-100, 0.2% gelatin (Millipore Sigma G7041)] supplemented with 2% donkey serum (Jackson Immunoresearch 102644-006) at room temperature for 2 h followed by an overnight rotation with mouse anti-MSP (Developmental Studies Hybridoma Bank, University of Iowa, United States; 4A5) (1:5) or chicken anti-GFP (Thermo Fisher A10262) (1:500) at 4°C in 0.2% gelatin solution supplemented with 0.5% donkey serum. Next day, samples were rinsed with PT Buffer followed by rotation in PT Buffer for 2 h at room temperature. Samples were incubated with goat anti-mouse Alexa Fluor 568 (Thermo Fisher A11031) (1:100) or goat anti-chicken Alexa Fluor Plus 647 (Thermo Fisher A32933) (1:1000) and rotated in the dark overnight at 4°C in 0.2% gelatin solution supplemented with 0.5% donkey serum. Next day, samples were washed with PT Buffer followed by rotation in PT Buffer for 1.5 h at room temperature in the dark. The DNA counterstain DAPI (Thermo Fisher D1306) was added and rotated for an additional 30 min followed by rinsing with PT Buffer. Worm samples were pelleted and VectaShield H1000 antifade mounting medium (Vector Laboratories) was added. Samples were imaged on a Leica DM5500 B microscope fitted with a Leica CTR5500 electronic box and a Hamamatsu Digital Camera C10600 ORCA R2 with maximally projected images of z-stacks optimized by the Leica LAS AF 3.1.0 software followed by quantification on ImageJ (NIH). Immunofluorescence was done using two to three independent biological replicates.

### 2.11 Expression of *osm-9*p*::gfp*

Imaging of *osm-9*p*::gfp* expression in ADL was assessed as previously done (Sims et al., 2016) except that animals were dye filled with either DiD (1,1′-dioctadecyl-3,3,3′,3′-tetramethylindodicarbocyanine, 4-chlorobenzenesulfonate salt, Thermo Fisher D7757) (for 2–4 h) or DiI (1,1′-dioctadecyl-3,3,3′,3′-tetramethylindocarbocyanine perchlorate, Sigma Aldrich 42364) (for 30 min) (Wallace et al., 2016).

### 2.12 Octanol avoidance assays

Avoidance assays to the odorant 1-octanol (TCI O0036) by 1-day old adults was done as detailed by Troemel et al. (1997). In short, square petri dishes (Fisher Scientific FB0875711A) containing 2% BD Difco Bacto agar (Fisher Scientific DF0140154) in 5 mM potassium phosphate, 1 mM CaCl_2_, and 1 mM MgSO_4_ was divided into six equally sized sectors (A-F) where the two most distal sectors (A and F) from the center served as the sites for the deposition of 1-octanol and the control (100% ethanol) with 1 μL 1 M NaN_3_ anesthetic pipetted equidistantly near the plate’s edge. One-day adults grown at 20°C in NGM plates seeded with OP50 were collected and washed with S-basal buffer (0.1 M NaCl, 0.05 M potassium phosphate pH 6.0) until the supernatant was clear followed by one rinse in dH_2_O. Worms (∼200) were placed onto the center (between sectors C and D) of the petri dish equidistantly to sectors A and F where 1 μL of 100% ethanol (sector F) and 1-octanol (sector A) was immediately pipetted. Excess liquid was rapidly removed using a kimwipe without disturbing the animals or puncturing the agar. Assays were placed an undisturbed location at room temperature for 1 h after which worms were assessed for their reaction to octanol using the following formula: Avoidance Index = (A + B)-(E + F)/N; where A, B, E and F are the number of animals in these sectors and N is the total number of animals in all the six sectors.

### 2.13 Ascr#3 avoidance assays

Avoidance of ascr#3 was determined off-food using 1-day old adults grown at 20°C on NGM plates seeded with OP50 and performed essentially as previously done (Sims et al., 2016) except that forward-moving adults were first tested for their reaction to a drop of benign M13 buffer (30 mM Tris-HCl pH 7.0, 100 mM NaCl, 10 mM KCl) deposited near their tail and migrating up to their nose. 10–12 adults that were indifferent upon encountering M13 buffer were then tested for their behavior when presented with 100 nM ascr#3 and 1 M glycerol. A backward-moving motion consisting of 1.5 body lengths in 4 s was considered an avoidance response. Only animals that were tested in the same timeframe were subjected to statistical comparisons to the wild-type N2 control.

### 2.14 Chemotaxis assays

Chemotaxis assays for the benzaldehyde and diacetyl attractants were performed on 1-day adults as described in (Bargmann et al., 1993; Hart, 2006). Assays plates consisting of 2% BD Difco Bacto agar (Fisher Scientific DF0140154) in 5 mM potassium phosphate, pH 6.0, 1 mM CaCl_2_, and 1 mM MgSO_4_ were prepared on 100 mm round petri dishes. Two marks (one for the attractant and one for the 100% ethanol control), 180^o^ degrees opposite each other, were indicated on the bottom of the plate near its edge where 1 μL of 1 M NaN_3_ anesthetic was deposited. Worms grown at 20°C on OP50-seeded NGM plates were collected and washed with S-buffer (0.1 M NaCl, 0.05 M potassium phosphate pH 6.0) until the supernatant was cleared of their bacteria food followed by one wash with dH_2_O. Pipette worms (∼200) onto the center of the assay plate, equidistantly from the marks and slightly off center. Excess liquid was quickly soaked off using a kimwipe without disturbing the animals or puncturing the agar. Immediately place 1 μL of the attractant and the ethanol on their respective marks, assay plate was closed with the lid and placed in an undisturbed location at room temperature for 1 h. Worms that were within a 0.5 cm radius of the spot where the attractant and the ethanol were deposited were then counted and the chemotaxis index was calculated with the following formula: Chemotaxis Index (CI) = (number worms at the attractant after 1 h - number worms at control ethanol after 1 h)/total # of worms. Chemotaxis indexes of +1.0 to −1.0 indicated perfect attraction or repulsion, respectively.

### 2.15 Brood size assays

Ten L4 larvae were placed individually onto 35 mm NGM plates seeded with OP50 and transferred daily until egg laying ceased at 20°C. Brood sizes were determined by counting their live progeny as previously described (Ow et al., 2018; Ow et al., 2021).

## 3 Results

### 3.1 Wild-type adult ADL neurons express germline-enriched genes

To identify additional genes expressed in ADL neurons that may be regulated by passage through dauer, we used fluorescence activated cell sorting (FACS) followed by RNA-Seq to characterize the transcriptome of ADL chemosensory neurons in 1-day old control (CON) and postdauer adults that have experienced dauer resulting from crowding (PD_Phe_). This experiment was performed using a strain that expressed an integrated extra-chromosomal array of *sre-1*p::*gfp* to facilitate isolation of ADL neurons (Supplementary Figure S1). Using an expression cutoff of 10 RPKM (reads per kilobase of transcript per million reads), we identified 5908 and 6375 genes that are expressed in control and PD_Phe_ ADL neurons, respectively (Supplementary Table S2). We compared our set of expressed genes with the ADL transcriptome profiled by the CeNGEN consortium (https://cengen.org) (7217 genes) that generated expression profiles of the 302 neurons comprising the *C. elegans* L4 larval nervous system in hermaphrodites (Taylor et al., 2021). The ADL CeNGEN dataset overlapped with 62% (3652 genes; *p* = 1.26e-514) and 65% (4129 genes; *p* = 6.30e-719) compared to our adult control and PD_Phe_ transcriptomes, respectively (Supplementary Table S3). Although there are significant similarities between our gene lists and the CeNGEN consortium, the difference in transcriptomes may be ascribed to procedural variances or developmental stages assayed. We also compared a previously described pan-neuronal transcriptome of 8436 genes collected from 1-day old adults with our dataset (Kaletsky et al., 2018). We found an 81% (4779 genes; *p =* 9.75e-1161) and 82% overlap (5240 genes; *p* = 3.71e-1396) with our control and PD_Phe_ gene lists, respectively (Supplementary Table S4). Based on these similarities, we conclude that our gene lists are an accurate representation of the ADL transcriptome.

**FIGURE 1:**
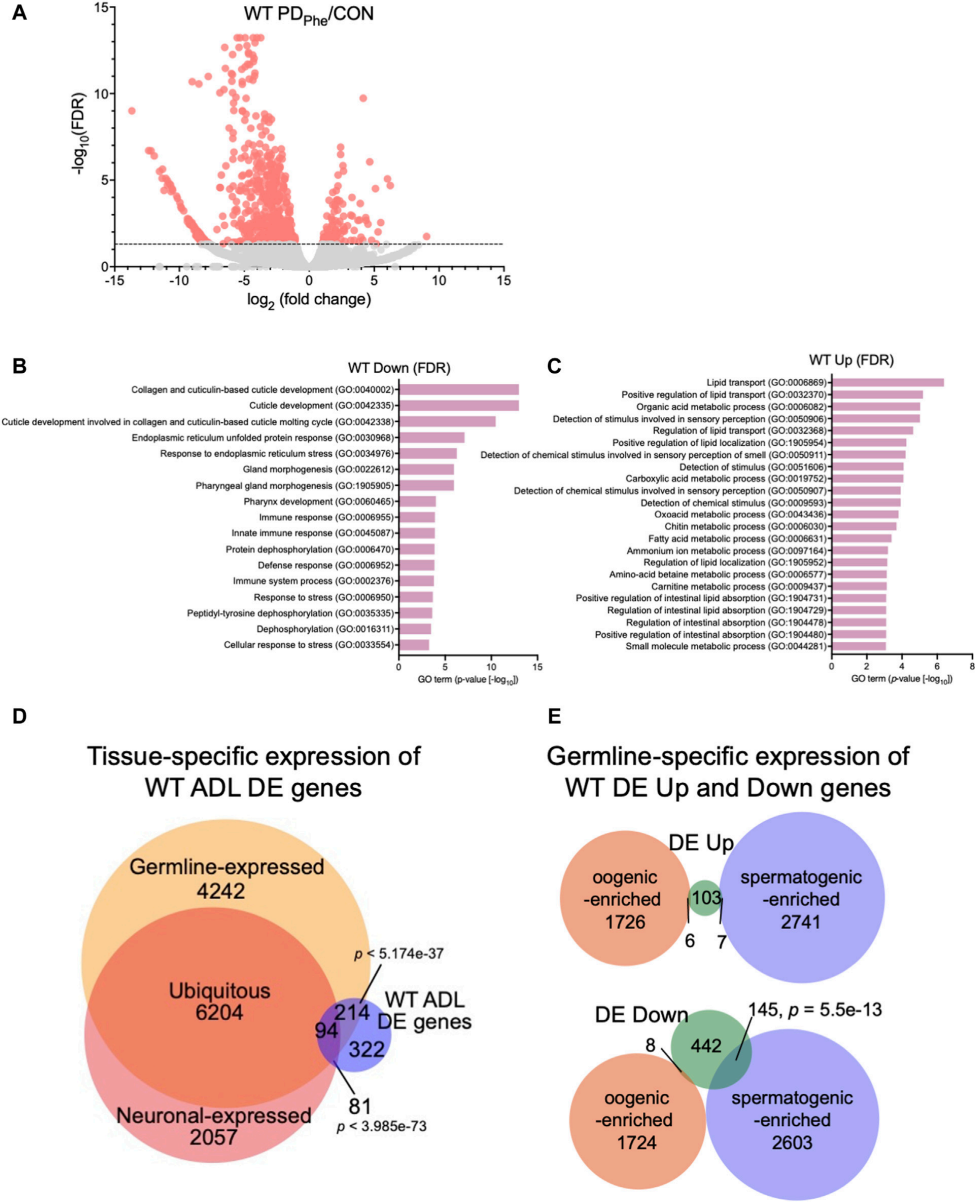
Wild-type adult ADL neurons express germline genes. **(A)** Volcano plot representing WT CON and PD_Phe_ adult ADL RNA-Seq data. Dotted line indicates FDR cutoff of *p* < 0.05; salmon dots represent mRNAs that are either significantly upregulated or downregulated in PD_Phe_ compared to CON. **(B,C)** GO terms of genes that are most significantly **(B)** decreased or **(C)** increased based on FDR < 0.05 in WT PD_Phe_ compared to WT CON. **(D)** Venn diagram comparing all differentially expressed genes in WT PD_Phe_ ADL neurons compared to WT CON ADL neurons (WT ADL DE) to germline (Ortiz et al., 2014) and neuronal expressed gene lists (Kaletsky et al., 2018). **(E)** Venn diagrams comparing spermatogenic- and oogenic-enriched (Ortiz et al., 2014) gene lists with wild-type differentially expressed up- (DE Up) and downregulated (DE Down) genes. *p*-values for Venn diagrams were determined using a hypergeometric distribution.

We next identified the set of differentially expressed (DE) genes between control and PD_Phe_ ADL neurons. Using an FDR <0.05 cutoff, we identified 116 and 595 genes that were up- and downregulated, respectively, between the two populations (Figure 1A; Supplementary Table S5). From here on, these genes will be called “WT DE Up” or “WT DE Down” to refer to wild-type PD_Phe_ adult genes that are differentially up- or downregulated, respectively, compared to their control counterparts. The *osm-9* gene was not present in this group due to its low expression levels, despite being previously validated as significantly downregulated in ADL PD_Phe_ neurons (Sims et al., 2016), suggesting that the genes we identified have robust gene expression changes following the dauer experience. GO term analyses of WT DE Down genes uncovered significant associations with functions related to collagen and cuticle development, endoplasmic reticulum (ER) stress, immune and stress response (Figure 1B; Supplementary Table S6), whereas WT DE Up genes were associated with lipid transport and function, stimuli response, oxidation-reduction process, and metabolic processes (Figure 1C; Supplementary Table S7). The WT DE Up genes include the G protein-coupled receptor (GPCR) genes *srh-281*, *sri-32*, *srw-89*, *srz-47*, *srh-204*, *srsx-7*, *srsx-6*, and *srw-23,* as well as insulin-like genes *ins-12* and *irld-15* (Supplementary Table S5). A comparison with CeNGEN showed that six out of eight of GPCRs and both insulin-like genes were expressed in ADL: *sri-32*, *srw-89*, *srz-47*, *srh-204*, *srsx-7*, *srsx-6*, *ins-12*, and *irld-15* (Taylor et al., 2021). Notably, *srh-281*, the most highly expressed GPCR gene in our PD_Phe_ gene list is solely expressed in ADL (Vidal et al., 2018), further highlighting the validity our ADL transcriptome (Supplementary Tables S3, S5).

Comparison of the overlap between the 711 WT DE genes with the pan-neuronal transcriptome from 1-day old adults (Kaletsky et al., 2018) found that only 24.6% of the differential expressed genes in ADL were previously shown to be expressed in neurons (175 genes; *p* = 3.99e-73) (Figure 1D; Supplementary Table S8). The limited overlap between these two gene sets indicates that the vast majority of differentially expressed genes in ADL neurons are not the previously identified genes that function in neuronal plasticity. However, comparison of the ADL DE gene list with a germline-specific gene list (Ortiz et al., 2014), showed that a significant proportion of 43% of ADL DE genes (308 genes; *p* = 5.17e-37) overlapped with genes known to be germline-expressed (Figure 1D; Supplementary Table S9). To further distinguish between the classes of germline genes that are in common between our ADL-derived gene list and germline-enriched genes, we compared our list of upregulated and downregulated genes with previously identified spermatogenic- and oogenic-enriched genes (Ortiz et al., 2014). For upregulated genes in ADL, 7 genes were in the spermatogenic-enriched gene list (2.3-fold under enriched; *p* = 0.008) while 6 genes were in the oogenic-enriched gene list (1.7-fold under enriched; *p* = 0.12) (Figure 1E; Supplementary Table S10). For the downregulated genes, 145 spermatogenic genes (1.8-fold enriched; *p* = 5.5e-13) and 8 oogenic genes (6.4-fold under enriched; *p* = 1e-14) overlapped with both gene lists (Figure 1E; Supplementary Table S10). The 145 downregulated spermatogenic enriched genes included genes encoding for two major sperm protein domain-containing genes *(msd-3/-4*) and 21 (*msp-3/-10/-19/-31/-36/-38/-45/-49/-50/-51/-55/-56/-64/-65/-77/-78/-79/-81/-113/-152*) of the 48 predicted *major sperm protein* genes expressed in *C. elegans* (http://www.wormbase.org, release version WS287) (Figure 1E; Supplementary Table S10). These comparisons indicate that ADL neurons express hundreds of genes that have been previously considered to be exclusively expressed in the germ line. While the number of oogenic genes expressed in ADL is greater that spermatogenic genes, the spermatogenic genes, such as MSPs, are specifically targeted for downregulation in postdauer ADL neurons similar to *osm-9* (Figures 1D,E; Supplementary Tables S9, S10). Collectively, these results indicate that a subset of germline genes are expressed in ADL neurons and are targeted for differential expression based early-life experiences.

### 3.2 NRDE-3 is required for differential expression of major sperm protein genes in ADL

**FIGURE 2:**
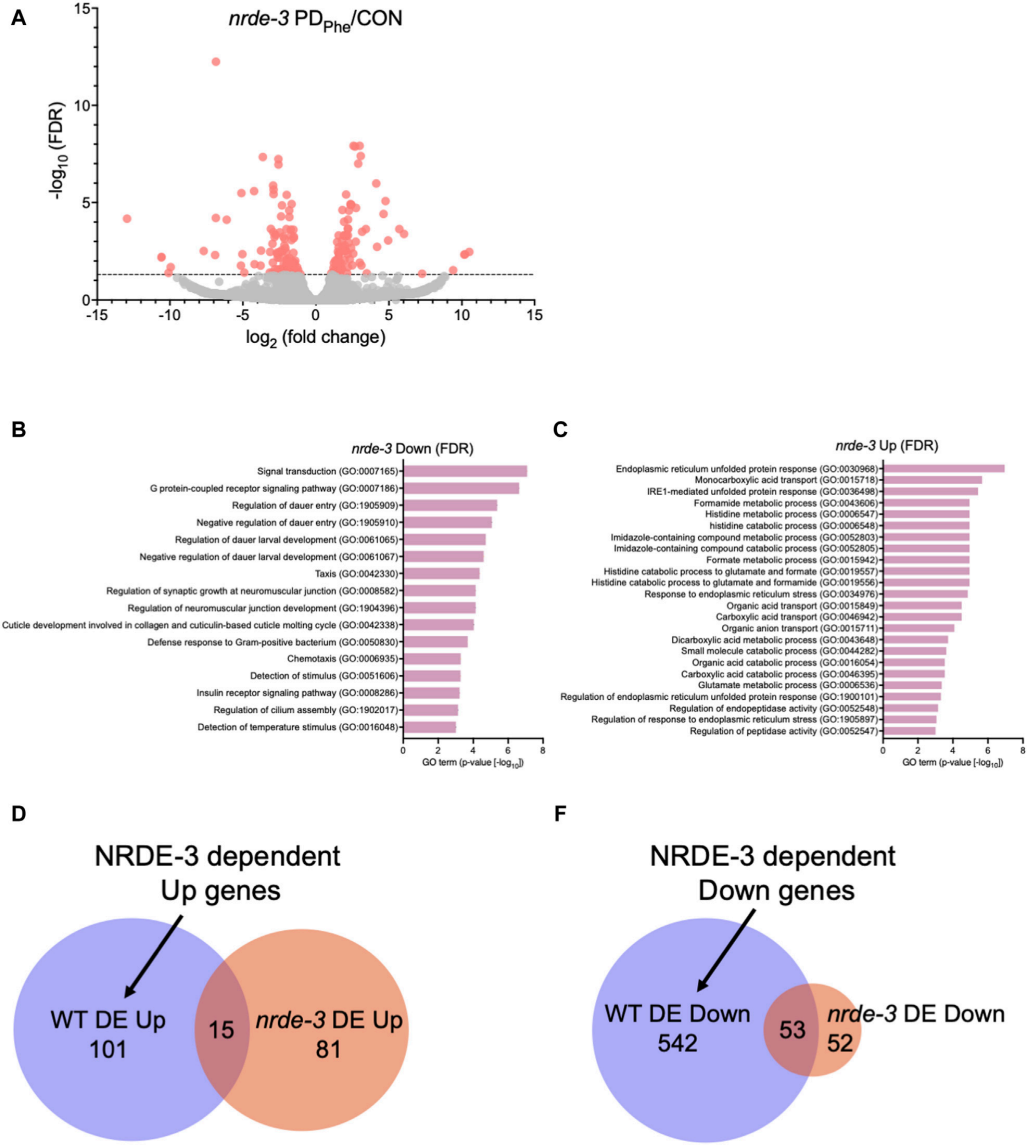
The NRDE-3 somatic nuclear RNAi pathway is required for differential expression of ADL genes. **(A)** Volcano plot representing *nrde-3(gg66)* CON and PD_Phe_ adult ADL RNA-Seq data. Dotted line indicates FDR cutoff of *p* < 0.05; red dots represent mRNAs that are either significantly up- or downregulated in PD_Phe_ compared to CON. **(B,C)** GO terms of genes that are most significantly **(B)** decreased or **(C)** increased based on FDR <0.05 in *nrde-3* PD_Phe_ compared to *nrde-3* CON. **(D,E)** Venn diagrams comparing WT and *nrde-3* gene lists for **(D)** DE Up and **(E)** DE Down genes.

We showed previously that *osm-9* downregulation in PD_Phe_ ADL neurons requires components of the somatic nuclear RNAi pathway, including NRDE-3, NRDE-1, and NRDE-4 (Sims et al., 2016). To examine whether the somatic nuclear RNAi complex plays a broader role in programming an animal’s mRNA transcriptome in response to early-life environmental experience, we set out to identify additional targets of NRDE-3 AGO by performing RNA-Seq of control and PD_Phe_ ADL neurons collected from the *nrde-3(gg66*) mutant strain. Using an expression cutoff of RPKM >10, we identified 6001 and 6377 genes that are expressed in the ADL neurons of control and PD_Phe_ *nrde-3* adults, respectively (Supplementary Table S11). Comparison of the transcriptomes from wild-type and *nrde-3* control ADL neurons resulted in a 77.7% similarity (5206 genes) (*p* = 5.17e-3045) (Supplementary Table S12). Similarly, the transcriptomes of the wild-type and *nrde-3* PD_Phe_ neurons shared an 87.5% similarity (5951 genes) (*p* = 4.07e-3939) (Supplementary Table S12). Differential expression analysis of ADL genes in the *nrde-3* mutants revealed 105 genes that are downregulated and have functions in signal transduction, G protein-coupled receptor signaling, dauer entry, and detection of stimuli (Figures 2A,B; Supplementary Table S13). In addition, we found 96 genes that are upregulated in *nrde-3* PD_Phe_ ADL neurons compared to controls with functions in endoplasmic reticulum stress response pathways, organic molecule transport, and histidine metabolism (Figures 2A,C; Supplementary Table S14).

Next, we identified which genes were dependent on NRDE-3, either directly or indirectly, for their differential expression in wild-type ADL neurons. First, we compared the 116 genes upregulated in wild-type PD_Phe_ ADL neurons with the 96 genes that are upregulated in *nrde-3* PD_Phe_ ADL neurons (Supplementary Tables S5, S15). We found only 15 genes overlapping between the two gene lists, indicating that 101 genes require NRDE-3 for their upregulation in PD_Phe_ ADL neurons in wild-type adults (Figure 2D; Supplementary Table S16). Amongst this gene set were 33 (32.7%) secreted protein genes, 26 (25.7%) transmembrane domain genes, 10 (9.9%) serpentine receptor genes involved in chemoreception, and 10 (9.9%) lipid metabolism genes (Suh and Hutter, 2012; Zhang et al., 2013). The lipid metabolism genes included the Rieske-like oxygenase *daf-36* important for cholesterol metabolism, Δ9 desaturase *fat-7* involved in lipid metabolism, and six vitellogenins, *vit-1/-2/-3/-4/-5/-6,* that have known functions to transport lipids into the germ line (Ow et al., 2021). We then compared the 595 downregulated ADL genes in wild-type PD_Phe_ adults to the 105 genes that are differentially downregulated in *nrde-3* mutants. We found 53 genes overlapping between the two gene lists, indicating that 542 downregulated genes in wild type ADL neurons were dependent on NRDE-3 function for their differential expression (Figure 2E; Supplementary Tables S5, S15, S17). Notably, sperm-expressed genes were members of this gene set, including 20 *msp* genes and the two PP1 phosphatases responsible for sperm development and motility, *gsp-3* and *gsp-4* (Wu et al., 2012). Additional genes in this group include 81 collagen genes (14.9%), 273 genes (50%) predicted to encode secreted proteins, 77 genes (14.2%) encoding transmembrane domain proteins, and 58 endoplasmic reticulum (ER) genes (10.7%) (Suh and Hutter, 2012), the latter of which include those in ER unfolded protein response and are amongst the most significantly upregulated genes present in *nrde-3* PD_Stv_ adults. Also included is the *odr-7* gene, required for the function of the AWA chemosensory neurons in a germline-dependent neural circuitry response to the odorant diacetyl during development (Sengupta et al., 1994; Fujiwara et al., 2016). Together, these results show that NRDE-3 plays a significant role in the regulation of genes in ADL neurons, particularly after passage through dauer due to crowding. The NRDE-3-dependent differentially expressed genes included those with functions related to fertility, reproduction, odor response, and fat metabolism, which are phenotypic traits that we have previously observed to be significantly altered in adults that experienced dauer (Sims et al., 2016; Ow et al., 2018; Ow et al., 2021).

### 3.3 A conserved upstream regulatory sequence is enriched for genes with NRDE-3 dependent expression changes

**FIGURE 3:**
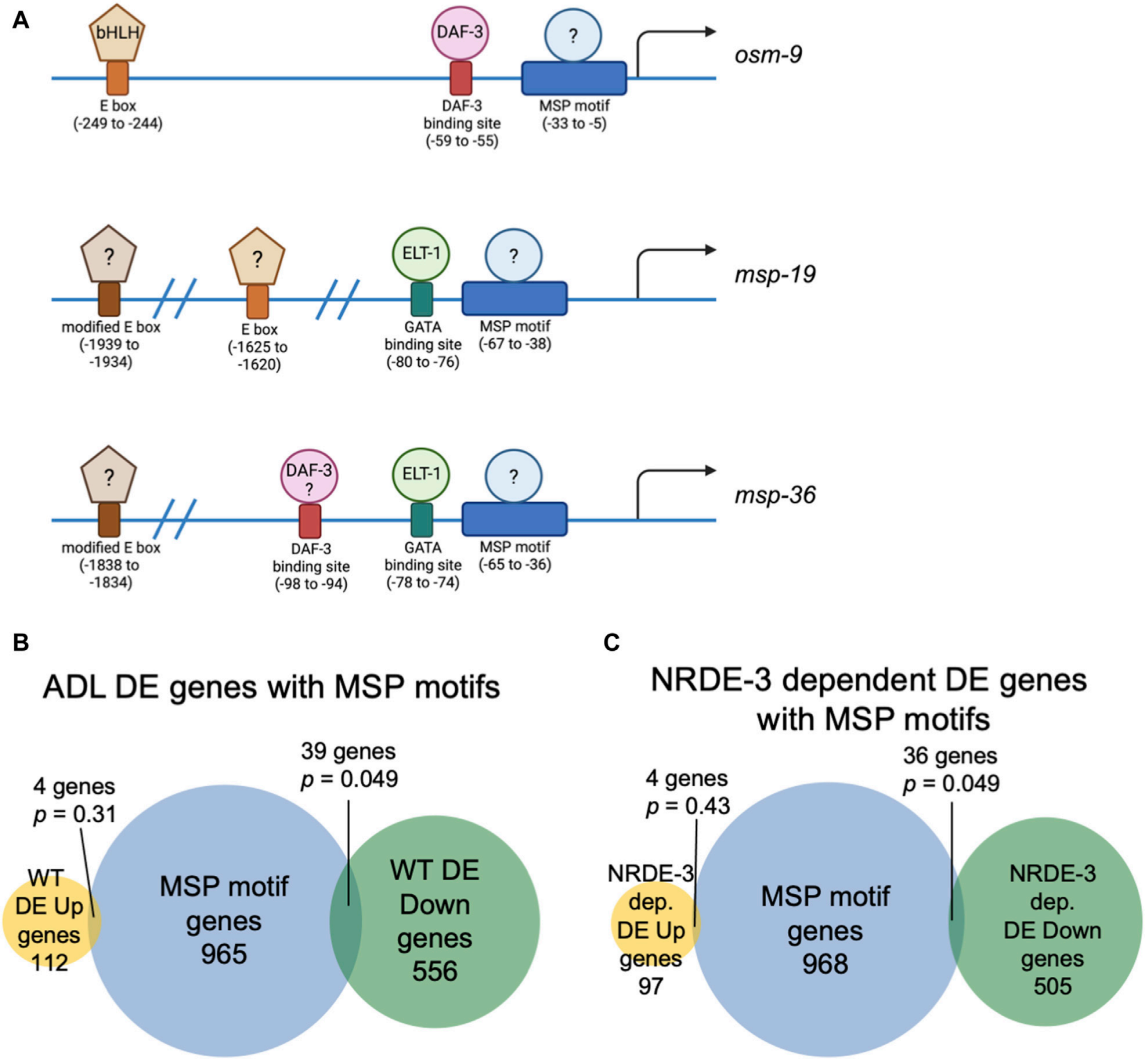
Genes with NRDE-3-dependent expression changes are enriched for a conserved sequence in their upstream regulatory regions. **(A)** Diagrams of upstream regulatory regions of *osm-9*, *msp-19*, and *msp-36* genes. Names of sequence motifs, location relative to translational start sites, and known interactors are indicated. **(B,C)** Venn diagrams comparing the set of genes containing an MSP motif in their 5′ UTRs with **(B)** all WT and **(C)** NRDE-3 dependent DE Up and DE Down gene lists. *p*-values were determined using a hypergeometric distribution.

In *C. elegans*, neuronal gene expression is modular in that the presence and arrangement of transcription factor binding sites in the upstream regulatory sequences can specify in which neurons that gene is transcribed (Stefanakis et al., 2015; Reilly et al., 2020; 2022; Hobert, 2021; Leyva-Díaz and Hobert, 2022). We next asked if ADL-expressed germline genes have the hallmarks of neuronal gene expression in their upstream regulatory sequences. *C. elegans* expresses over 40 MSP genes, and 20 of these genes exhibited differential expression in our ADL gene set (Supplementary Table S10). Previously, an ADL-enhancer element consisting of the CASCTG E-box motif (S=C or G) was found by analyzing promoters of ADL-expressed chemoreceptor genes (McCarroll et al., 2005). A similar motif of CACGTG was identified as overrepresented in *msp* promoter regions (McCarroll et al., 2005). While the modified E-box CACGTG motifs were present in 7 of the MSP genes expressed in ADL (*msp-19, -36, -55, -56, -64, -142, -152*), we also identified 1 to 3 copies of the ADL specific CASCTG E-box motif in the 5’ UTR sequences of 11 MSP genes (*msp-10, -19, -31, -38, -45, -50, -51, -55, -77, -81, -113*) (Figure 3A). These observations underscore the possibility that *msp* expression in ADL could be dictated at the transcriptional level for ADL specification.

We next asked whether the differential regulation of gene expression in ADL is also dependent upon upstream regulatory sequences. We previously identified a *cis*-acting motif located in the *osm-9* promoter, referred to as the “PD motif”, that was required for its transcriptional silencing in ADL of PD_Phe_ adults (Sims et al., 2016). This motif is composed of a DAF-3/SMAD binding site (GTCTA) and a ∼30 base pair conserved sequence (CT[A/T]TAA[A/T]TTN_0-3_[A/C]AN_0-2_TTTTG[C/T]CATAA[T/A]C[T/C]) that is responsive to TGF-ß signaling (Sims et al., 2016; Pekar et al., 2017). While >6000 genes possess the DAF-3 binding site in their upstream regulatory regions, 1008 genes contain the conserved sequence, and 116 genes the full PD motif (Supplementary Table S18). Interestingly, nine genes, in addition to *osm-9*, that were downregulated in WT postdauer ADL neurons compared to controls contain a full PD motif: *msd-3*, *msp-36*, *msp-51*, *msp-65*, *msp-81*, *msp-79*, *col-133*, B0205.14, and T16G1.2 (Supplementary Tables S5, S18). Since five of these genes were MSP genes, we next examined the overlap of differentially expressed genes in ADL and the list of genes containing just the conserved sequence in their upstream regulatory regions. For all genes differentially expressed in ADL, we found an overlap of 4 and 39 genes for the up- and downregulated gene lists, respectively (Figure 3B). A comparison of genes that exhibit NRDE-3-dependent differential expression in ADL with the list of genes with conserved sequences revealed a similar overlap of 4 and 36 genes for the up- and downregulated gene set, respectively (Figure 3C; Supplementary Tables S16–S18). The downregulated genes with conserved sequences included 19 MSP genes (*msp-3, -10, -19, -31, -36, -38, -45, -49, -50, -51, -55, -56, -65, -77, -78, -79, -81, -113, -152*). Future work will determine if the downregulation of MSP genes in postdauer ADL neurons may be attributed to binding of an unidentified factor to the conserved sequence in their promoter (Figure 3A). Given the enrichment of the conserved sequence in MSP gene regulatory sequences, we will henceforth refer to this ∼30 base pair conserved sequence element in the PD motif as the “MSP motif”.

### 3.4 Major sperm proteins are expressed in ADL neurons

**FIGURE 4:**
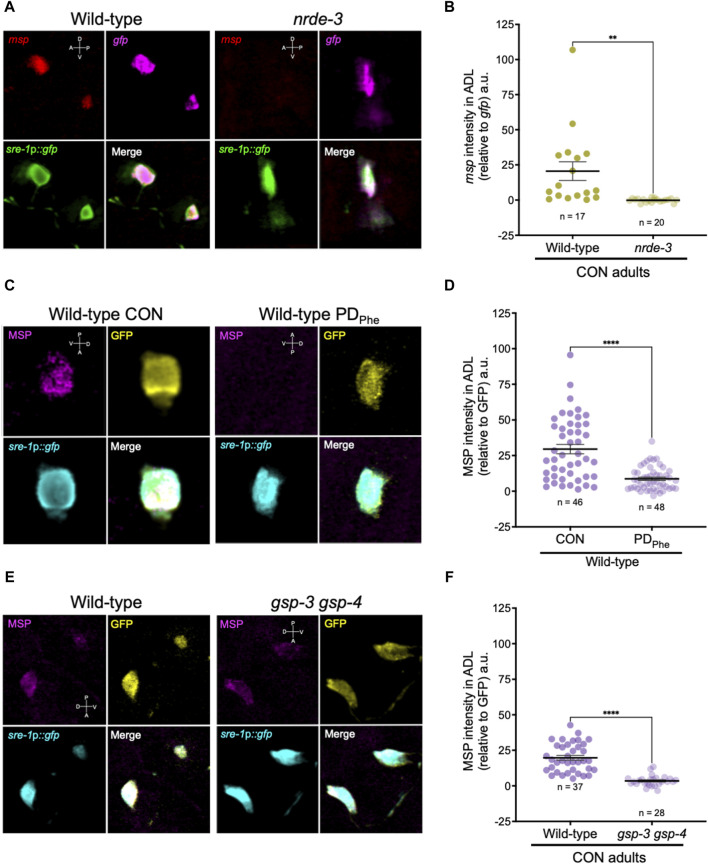
Major sperm proteins are expressed in ADL neurons. **(A)** Representative images of smFISH of ADL neurons with probes against *msp* (red) and *gfp* (magenta) mRNAs using 1-day old wild-type and *nrde-3(gg66)* CON adults. *sre-1*p*::gfp* transgene fluorescence shown in green. Images of animal heads shown in Supplementary Figure S2. **(B)** Quantification of *msp* relative to *gfp* smFISH intensity in ADL; a.u. are arbitratry units. ** *p* < 0.01, Student’s t-test of three independent experiments. **(C)** Representative images of immunostainings using antibodies against MSP (magenta) and GFP (yellow) on 1-day old wild-type CON and PD_Phe_ adults. *sre-1*p*::gfp* transgene fluorescence shown in cyan. Images of animal heads are shown in Supplementary Figure S3. **(D)** Quantification of MSP immunofluorescence intensity relative to the intensity of GFP immunofluorescence. **** *p* < 0.0001, Student’s t-test of two independent experiments. **(E)** Representative images of immunostainings using antibodies against MSP (magenta) and GFP (yellow) on 1-day old wild-type and *gsp-3(tm1647) gsp-4(y148)* CON adults. *sre-1*p*::gfp* transgene fluorescence shown in cyan. Images of animal heads are shown in Supplementary Figure S4. **(F)** Quantification of MSP immunofluorescence intensity relative to the intensity of GFP immunofluorescence. **** *p* < 0.0001, Student’s t-test of three independent experiments. Number of animals are indicated by n. D, V, A, P represent the dorsal, ventral, anterior, and posterior orientation of the animal. Additional data included in Supplementary Table S20.

Since the expression of spermatogenic genes in wild-type ADL neurons was unexpected, we sought to validate the expression of *msp* genes in ADL. First, we performed single molecule fluorescence *in situ* hybridization (smFISH) to detect the presence of *msp* mRNAs in wild-type and *nrde-3* control adults carrying the *sre-1*p*::gfp* transgene expressed in ADL neurons (Supplementary Figure S1). Our RNA-Seq results showed that wild-type control adults express *msp-19* at an 11-fold higher level than in *nrde-3* control ADL neurons (RPKM = 129.91 vs. RPKM = 11.61, respectively) (Supplementary Table S19); thus, we chose *msp-19* as the template for smFISH probes design. However, because of the high sequence homology of the *msp* genes (Kasimatis and Phillips, 2018), it is likely that most, if not all, *msp* mRNAs were detected using our probes. Using smFISH, we detected *msp-19* mRNAs in wild-type control ADL neurons (Figures 4A,B; Supplementary Figure S2). Consistent with our RNA-Seq results, the *msp-19* signal was significantly decreased in *nrde-3* mutants, validating that NRDE-3 is required for the expression of *msp* mRNA in control ADL neurons (Figures 4A,B; Supplementary Figure S2). As a positive control, we also performed smFISH with probes detecting *gfp* mRNA, and we observed no significant differences in *gfp* fluorescence intensity in ADL neurons of wild-type and *nrde-3* control adults (Figure 4A; Supplementary Figure S2).

Next, we examined whether the *msp* mRNAs were being translated by performing indirect immunofluorescence in ADL neurons of wild-type control and PD_Phe_ adults expressing the *sre-1*p*::gfp* transgene. Using a monoclonal antibody that recognizes MSP-19, -31, -40-, 45, -50, -51, -53, -59, -61, -65, -81, -113, and -142 (Developmental Studies Hybridoma Bank, University of Iowa, United States; 4A5), we found that control ADL neurons expressed detectable MSPs, and PD_Phe_ ADL neurons exhibited a significant downregulation of MSP (Figures 4C,D; Supplementary Figures S3A, S3C). As a positive control, we also performed indirect immunofluorescence with anti-GFP antibody and observed similar levels of GFP fluorescence intensity between wild-type control and PD_Phe_ ADL neurons. As an additional control, we verified that MSP staining was visible in both control and PD_Phe_ adult gonads (Supplementary Figures S3B, S3D). Overall, these results demonstrate that MSPs are expressed in ADL neurons of continuously developed hermaphrodites in a NRDE-3 dependent manner and are downregulated specifically in ADL neurons of adults that experienced crowding-induced dauer.

### 3.5 Sperm-expressed phosphatase is required for attraction and avoidance behaviors

After verifying the expression of germline genes in ADL neurons, we next sought to determine what role they play in ADL function. The *msp* genes comprise a 48-membered gene family with highly conserved sequences that encode 14 kilodalton (kDa) proteins (Scott et al., 1989). MSPs are the most abundant protein in nematode sperm, comprising ∼17% of their total protein and concentrating up to 3-fold in their sole locomotive structure, the pseudopod, compared to the cell body (Klass and Hirsh, 1981; Ward and Klass, 1982; King et al., 1992; Smith, 2014). Nematode sperm lack a flagellum, and MSPs substitute for a function that would otherwise be performed by structural proteins such as actin and myosin by forming a pseudopod (Nelson and Ward, 1981; Nelson et al., 1982; Roberts and Stewart, 1995; 2000; Bottino et al., 2002). MSPs polymerize into filaments at the leading edge of pseudopod in mature spermatozoa and disassemble at its trailing edge in an assembly-disassembly mechanism results in a treadmill-like form of locomotion (Sepsenwol et al., 1989; Sepsenwol and Taft, 1990; Roberts and King, 1991; King et al., 1994; Smith, 2014). In addition to enabling sperm movement, MSPs are also extracellular signaling molecules secreted by sperm that function as hormones to trigger oocyte maturation and ovulation, despite them lacking sequence motifs associated with secretory proteins (Miller et al., 2001; 2003; Kosinski et al., 2005). Although originally discovered in nematodes, the phylogenetic scope of proteins with MSP-like domains extends from plants to humans (Burke and Ward, 1983; Skehel et al., 1995; Laurent et al., 2000; Skehel et al., 2000).

**FIGURE 5:**
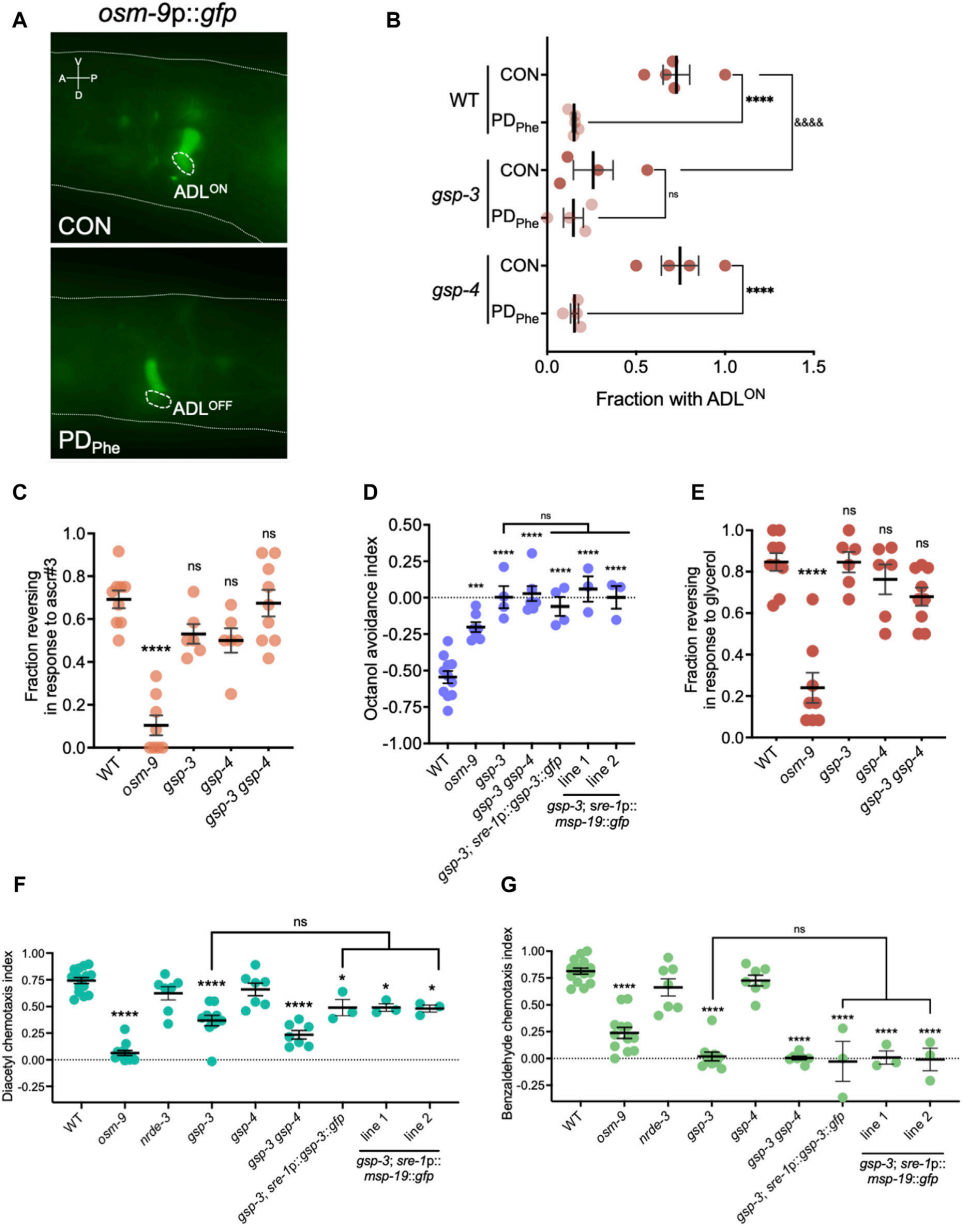
Sperm genes are required for olfactory behaviors. **(A)** Images of *osm-9*p*::gfp* fluorescent expression in WT control and postdauer adults. Dashed ovals indicate location of ADL neurons. The dashed line delineates a part of the head. A, V, P, and D indicate the anterior, ventral, posterior, and dorsal orientation of the animal. **(B)** Fraction of animals with ADL^ON^ status in wild-type, *gsp-3(tm1647)*, and *gsp-4(y148)* CON and PD_Phe_ 1-day adults expressing the *osm-9*p*::gfp* transgene reporter. **** and ^&&&&^ *p* < 0.0001; ns = not significant (Fisher’s exact test). Dots represent independent biological trials. **(C)** Fraction of 1-day old wild-type, *osm-9* *(ky10)*, *gsp-3(tm1647)*, *gsp-4(y148),* and *gsp-3(tm1647) gsp-4(y148)* CON adults reversing in ascr#3 avoidance behavior assays. **** *p* < 0.0001; one-way ANOVA with Dunnett’s *post-hoc* test. **(D)** Avoidance index of 1-day old wild-type, *osm-9* *(ky10)*, *gsp-3(tm1647)*, and *gsp-3(tm1647) gsp-4(y148)* CON adults in response to 1-octanol. Additional strains include expression of GSP-3 (*gsp-3*; *sre-1*p::*gsp-3*::*gfp*) or MSP-19 (*gsp-3*; *sre-1*p::*msp-19*::*gfp* lines 1 and 2) in ADL neurons in *gsp-3* mutants. *** *p* < 0.001, **** *p* < 0.0001, one-way ANOVA with Tukey’s *post-hoc* test. **(E)** Fraction of 1-day old wild-type, *osm-9* *(ky10)*, *gsp-3(tm1647)*, *gsp-4(y148),* and *gsp-3(tm1647) gsp-4(y148)* CON adults reversing upon exposure to 1M glycerol. **** *p* < 0.0001; one-way ANOVA with Dunnett’s *post-hoc* test. **(F,G)** Chemotaxis indexes of 1-day old wild-type, *osm-9*(ky10), *nrde-3(gg66)*, *gsp-3(tm1647)*, *gsp-4(y148),* and *gsp-3(tm1647) gsp-4(y148)* CON adults in response to **(F)** diacetyl and **(G)** benzaldehyde. Additional strains include expression of GSP-3 (*gsp-3*; *sre-1*p::*gsp-3*::*gfp*) or MSP-19 (*gsp-3*; *sre-1*p::*msp-19*::*gfp* lines 1 and 2) in ADL neurons in *gsp-3* mutants. * *p* < 0.05, **** *p* < 0.0001; one-way ANOVA with Tukey’s *post-hoc* test. ns = not significant. Each dot in Figures 5B–F represents an independent biological trial. Additional data included in Supplementary Table S20.

Mature sperm are considered transcriptionally and translationally dormant on account of their extremely compacted protamine-bound haploid genome and cytoplasmic shedding during the terminal stage of spermatogenesis; thus, mechanisms involving post-translational modifications are important for regulating fertility (Hecht, 1998). In mammals, the serine/threonine PP1 phosphatases play critical roles in modulating sperm development and locomotion (Fardilha et al., 2011; Ferreira et al., 2022). *C. elegans* has two serine/threonine PP1 phosphatases, GSP-3 and GSP-4, which are required for MSP filament disassembly, and a double mutation in *gsp-3* and *gsp-4* results in sterility due to chromosome segregation defects and sperm immobility (Wu et al., 2012). Because of the technical difficulty to inactivate ADL-expressed *msp* genes simultaneously, we first investigated the potential of MSP function in ADL by characterizing the behavioral phenotypes of *gsp-3(tm1647)* and *gsp-4(y148)* mutants. Like the *msp* genes, *gsp-3* and *gsp-4* are only expressed in wild-type control ADL neurons in a NRDE-3 dependent manner (Supplementary Table S17). First, we examined MSP levels in a *gsp-3 gsp-4* double mutant strain expressing the *sre-1*p::*gfp* transgene and found that MSPs continue to be detected in ADL neurons, albeit at significantly lower levels than wild-type adults (Figures 4E,F; Supplementary Figures S4A, S4C). Previous work showed that GSP-3 and GSP-4 do not affect the transcription of *msp* genes in sperm (Supplementary Figures S4B, S4D) (Wu et al., 2012); thus, the observed decrease in MSP in the *gsp-3 gsp-4* mutant strain may be due to their effects on stability of the protein. Next, we asked whether GSP-3 and GSP-4 play a role in regulating the expression of additional genes by examining the fraction of ADL neurons that exhibit GFP due to expression an *osm-9*p*::gfp* transcriptional reporter transgene. We showed previously that in wild-type adults carrying the *osm-9*p*::gfp* transgene, GFP was expressed in ADL neurons (ADL^ON^) of control adults but was significantly downregulated in PD_Phe_ adults (ADL^OFF^), while the expression in AWA neurons remained unaffected (Figures 5A,B) (Sims et al., 2016). While *gsp-4* control and PD_Phe_ adults exhibited similar GFP expression as wild-type ADL neurons, *gsp-3* control adults had a significant reduction of animals expressing GFP, resulting in the loss of *osm-9*p*::gfp* expression difference between wild-type control and PD_Phe_ ADL neurons (Figure 5B). Our results are consistent with GSP-3, potentially through the action of MSPs, being required to regulate gene expression in ADL neurons of continuously developed hermaphrodites.

Next, we sought to determine if the *osm-9* gene expression changes in *gsp-3* mutants altered ADL functions. Avoidance of the pheromone component ascr#3 by hermaphrodites is an OSM-9-dependent and ADL-mediated behavior, and disruption of OSM-9 levels or function result in failure to avoid ascr#3 (Jang et al., 2012; Sims et al., 2016). To examine whether *gsp-3* mutants exhibit altered ascr#3 avoidance, we examined the fraction of *gsp-3*, *gsp-4,* and *gsp-3 gsp-4* double mutant control adults exhibiting an avoidance response to ascr#3. As expected, the wild-type control exhibited avoidance and the *osm-9(ky10)* mutant showed reduced avoidance in response to ascr#3 (Figure 5C) (Jang et al., 2012). The *gsp-3* and *gsp-4* single mutant strains showed a small, but non-significant, decrease in their ascr#3 avoidance response, while the *gsp-3 gsp-4* double mutant showed no significant difference compared to wild type (Figure 5C). These results suggest that GSP-3 and GSP-4 do not play a major role in regulation of ascr#3 avoidance behavior.

To further explore the function of ADL in the *gsp-3* mutants, we tested the avoidance of volatile odorant, 1-octanol, a behavior mediated by the ADL, ASH, and AWB neurons in the absence of food (Chao et al., 2004). In the presence of 1-octanol, the wild-type controls exhibited the expected avoidance behavior (negative index indicates avoidance), which was significantly reduced in the *osm-9* mutant strain (Figure 5D). Interestingly, the avoidance responses of the *gsp-3* and *gsp-3 gsp-4* mutant strains were eliminated (Figure 5D), suggesting that the function of other neurons may also be affected in the *gsp-3* mutants. To test this possibility, we measured avoidance to 1 M glycerol, which is an OSM-9-dependent behavior mediated by the ASH sensory neurons required for avoidance behavior in response to volatile stimuli, mechanical stimulation, or osmotic shock (Kaplan and Horvitz, 1993; Troemel et al., 1995; Colbert et al., 1997; Hilliard et al., 2005). Again, the wild-type controls and *osm-9* mutants exhibited the avoidance and lack of avoidance, respectively, that was expected in response to glycerol (Figure 5E). However, neither the *gsp-3* or *gsp-4* single mutants or the *gsp-3 gsp-4* double mutant exhibited significant defects in glycerol avoidance compared to wild type (Figure 5E). These results suggest that GSP-3 phosphatase activity is required specifically for octanol avoidance mediated by nociceptive neurons.

ADL and ASH sensory neurons are closely related in their avoidance functions and synaptic connections (Cook et al., 2019). To test if other neuron classes require GSP-3 and GSP-4 for their function, we examined the chemotaxis behavior of the *gsp-3* and *gsp-4* single and *gsp-3 gsp-4* double mutant strains in response to attractive odorants. Benzaldehyde and diacetyl are volatile odorants whose attraction to worms is mediated by the AWC and AWA neurons, respectively (Bargmann et al., 1993). As previously observed, wild-type adults showed robust attraction to diluted benzaldehyde (5/1000 dilution) and diacetyl (1/1000 dilution) (Colbert et al., 1997). In our assays, this attraction was abrogated by a mutation in the *osm-9(ky10)* gene, although OSM-9-dependent attraction to diluted benzaldehyde has not been reported previously (Figure 5F) (Colbert et al., 1997). We also tested *nrde-3(gg66)* mutant adults, which exhibited a small, but not significant, decrease in chemotaxis compared to wild-type adults. In contrast, the *gsp-3* and *gsp-3 gsp-4* mutant strains exhibited significant decreases in both diluted benzaldehyde and diacetyl attraction, while the *gsp-4* mutant showed a similar chemotaxis index as wild type (Figure 5F). Overall, these results indicate that sperm-expressed serine/threonine PP1 phosphatase GSP-3 is required for both avoidance and attraction behaviors mediated by different sensory neurons in adult hermaphrodites.

We next tested the hypothesis that GSP-3 is required cell non-autonomously for chemosensory behaviors by regulating the secretion of MSPs from ADL neurons. In the gonad, *gsp-3 gsp-4* double mutants have abnormal MSP localization in sperm and decreased MSP secretion promoting ovulation compared to wild-type animals (Wu et al., 2012). Our smFISH and immunofluorescence experiments suggest that MSPs are expressed only in ADL neurons (Supplementary Figures S2–S4); therefore, their secretion from ADL may impact the function of neighboring neurons. ADL, ASH, AWB, AWC, and AWA sensory neurons are located together in the same glial-like, amphid structure in the worm head (Ward et al., 1975). To test our hypothesis, we constructed a strain carrying a transgene that rescued the expression of *gsp-3* in ADL neurons in a *gsp-3(tm1647)* mutant background (*gsp-3*; *sre-1*p::*gsp-3*::*gfp*). We compared the octanol avoidance, diacetyl attraction, and benzaldehyde attraction indexes exhibited by this strain to the *gsp-3* mutant. For all three behaviors, we found that the expression of GSP-3 in ADL neurons was not sufficient to alter the behavior of the *gsp-3* mutants (Figures 5D,F,G). Next, we tested the hypothesis that decreased levels of MSPs in the ADL neurons of *gsp-3 gsp-4* mutants may play a role in altered chemosensory behaviors (Figures 4E, F). To test this possibility, we created a strain that carries an extrachromosomal array with a transgene that over-expresses *msp-19* under an ADL-specific promoter in the *gsp-3(tm1647)* background (*gsp-3*; *sre-1*p::*msp-19*::*gfp*). Using this strain, we tested the octanol avoidance, diacetyl attraction, and benzaldehyde attraction behaviors compared to *gsp-3* mutants alone. Similar to the ADL-specific rescue of GSP-3, we found that over-expression of MSP-19 in ADL was also not sufficient to alter the behavior of the *gsp-3* mutants (Figures 5D,F,G). Together, these results indicate that GSP-3 does not act in ADL to regulate the behavioral functions of other amphid neurons, suggesting the possibility that sperm genes may be expressed more widely in the *C. elegans* nervous system.

### 3.6 Loss of ADL neuronal function affects fecundity

Communication between neurons and the germ line is well documented in mammals, *Drosophila*, and *C. elegans* (Yoon et al., 2005; Häsemeyer et al., 2009; Hill and Elias, 2018; Wang et al., 2019; Gaddy et al., 2021). For example, in *C. elegans*, chemotaxis behavior in response to diacetyl by the activities of the AWA olfactory neurons and their downstream AIB and AIB interneurons is dependent on larval germline proliferation (Fujiwara et al., 2016). The neuronal activity of the ASJ temperature-sensing neurons is affected by a number of sperm genes, including *gsp-4* (Sonoda et al., 2016). The expression of *daf-7*/TGF-ß in ASI neurons promotes *lag-2* expression and activation of the GLP-1/Notch receptor to maintain the stem cell niche in the gonadal distal tip cells (Pekar et al., 2017). The detection of social pheromones by the worm nervous system directs the timing of post-embryonic germline development in the next-generation (Perez et al., 2021). Ectopic germline differentiation in worms due to endoplasmic reticulum (ER)-induced stress response is suppressed by a neuronal-germline circuit mediated by serotonin (Levi-Ferber et al., 2021). The male-enriched ascr#10 (asc-C9) sex pheromone, which serves in hermaphrodite attraction and mating success, also functions to shorten the lifespan and delay the reproductive senescence of its hermaphroditic mating partner in an ADL-dependent manner (Aprison and Ruvinsky, 2022).

**FIGURE 6:**
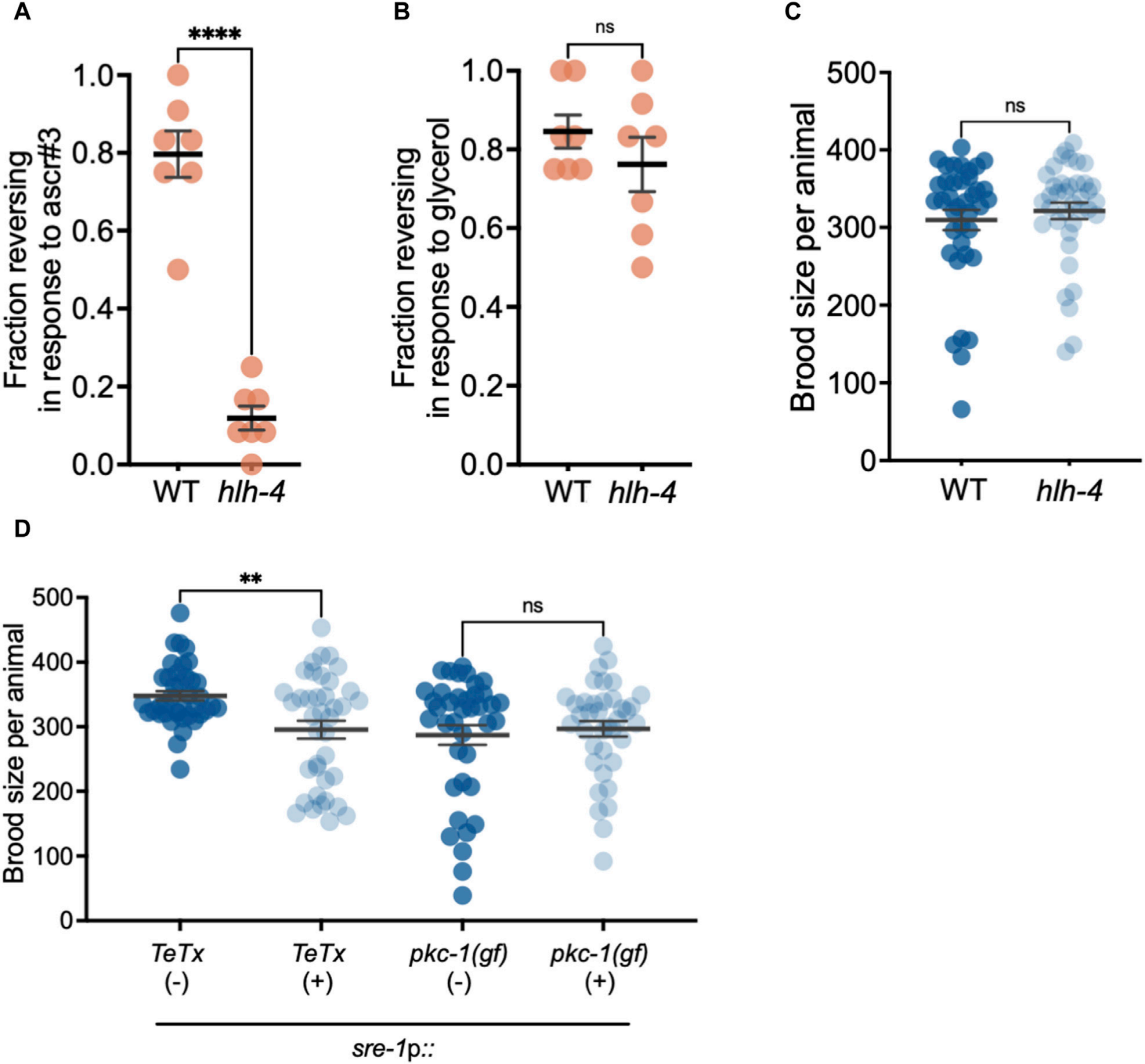
ADL chemosensory neurons contribute to fecundity. **(A,B)** Fraction of 1-day old wild-type and *hlh-4(tm604)* CON adults reversing in response to **(A)** ascr#3 and **(B)** 1M glycerol. **** *p* < 0.0001, Student’s t-test. Dots represent independent biological trials. **(C)** Brood size assays of wild-type and *hlh-4(tm604)* CON adults. ns = not significant; Student’s t-test. Data represents three independent biological replicates. **(D)** Brood size assays of CON adults with (+) or without (−) extra-chromosomal arrays consisting of tetanus toxin (TeTx) or a gain-of-function constitutively active protein kinase C isoform gene (*pkc-1 (gf)*) under the control of the *sre-1* promoter (*sre-1*p). ** *p* < 0.01, ns = not significant; Student’s t-test. Data represents three independent biological trials. Additional data included in Supplementary Table S20.

Based on these observations, we asked whether the expression of germline genes in ADL could influence germline development in a manner that would affect fecundity. During hermaphrodite germline development, mitotically dividing germline stem cells develop into sperm during the L4 larval stage, then switch to oocyte production during adulthood (L’Hernault, 2006). Therefore, the brood size of a *C. elegans* hermaphrodite resulting from self-fertilization is sperm-limited (Byerly et al., 1976; Ward and Carrel, 1979; Kimble and Ward, 1988). To examine whether ADL influences hermaphrodite fecundity, we measured the brood size of hermaphrodites that have a loss of ADL cellular identity or function. The *hlh-4* gene encodes a helix-loop-helix (bHLH) transcription factor that is required for ADL neuron fate and promotes expression of olfactory receptors that drive ADL-specific functions as a nociceptive sensory neuron (Masoudi et al., 2018). First, we verified that ADL identity is disrupted in *hlh-4(tm604)* mutants by showing that *hlh-4* adults were severely defective in ADL-dependent ascr#3 avoidance, but they were unaffected in ASH-dependent glycerol avoidance behaviors (Figures 6A,B). However, loss of ADL identity did not significantly impact the fecundity of *hlh-4* mutant hermaphrodites compared to wild type (Figure 6C).

Since HLH-4 is required for ADL neuronal identity but not for ADL birth (Masoudi et al., 2018), we next tested whether ablating ADL function would affect hermaphrodite fecundity. Expression of an extrachromosomal array expressing the light chain of the tetanus toxin (TeTx) under the control of the *sre-1* promoter has been shown to effectively eliminate ADL neuronal function by preventing chemical synapses (Jang et al., 2012). The neurotoxin TeTx is a zinc protease that specifically cleaves synaptobrevin, an essential synaptic vesicle membrane protein, and inhibits neurotransmitter release (Schiavo et al., 1992). We therefore asked whether blocking synaptic transmission in ADL would affect hermaphrodite reproduction by comparing the brood size of adults expressing the *TeTx* gene under the control of the *sre-1* promoter (*sre-1*p*::TeTx*) to adults of the same generation that lost the extrachromosomal array (Jang et al., 2012). We found that adults expressing the *TeTx* transgene exhibited a significant decrease in brood size compared to adults compared to the controls (Figure 6D). If ADL function is required to promote fecundity, we next asked whether hyperactive ADL would increase the brood size beyond controls. We increased ADL synaptic output by expressing an extrachromosomal array with a transgene carrying a constitutively active, *gain-of-function* allele of the protein kinase C isoform that promotes neuronal synapses, *pkc-1(gf)* (Okochi et al., 2005; Sieburth et al., 2007; Tsunozaki et al., 2008; Macosko et al., 2009; Jang et al., 2012). We observed no effect on brood size between adults carrying the array expressing *pkc-1(gf)* and adults from the same generation that lost the *pkc-1(gf)* transgene (Figure 6D). These results suggest that while eliminating ADL synaptic transmission slightly decreases hermaphrodite brood size, the germ line could be buffered from the consequences resulting from over-active ADL chemical synapses (Marchal and Tursun, 2021).

### 3.7 Germ cells are not required for ascr#3 avoidance behavior

**FIGURE 7:**
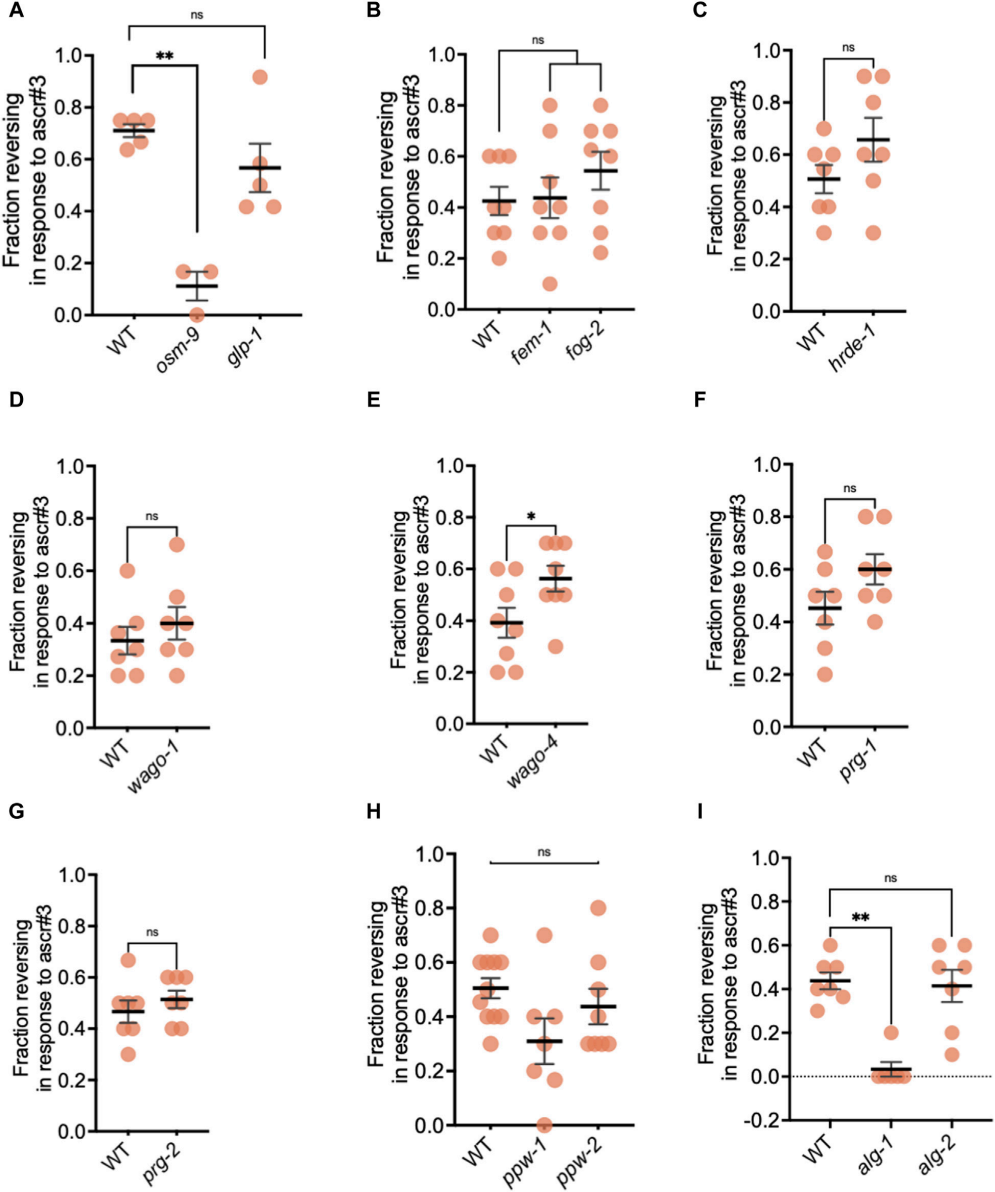
Endogenous small RNAi pathways mediate ascr#3 avoidance response. **(A–I)** Fraction of 1-day old CON adults reversing in response to ascr#3 for **(A)** wild-type, *osm-9* *(ky10)*, *glp-1(q224)*; **(B)** wild-type, *fem-1(hc17)*, *fog-2(q71)*; **(C)** wild-type*, hrde-1(tm1200)*; **(D)** wild-type*, wago-1(ok1074)*; **(E)** wild-type*, wago-4(tm1019)*; **(F)** wild-type, *prg-1(tm872)*; **(G)** wild-type N2*, prg-2(tm1094)*; **(H)** wild-type*, ppw-1(pk2505)*, *ppw-2(tm1120);* and **(I)** wild-type*, alg-1(gk214)*, *alg-2(ok304)* strains. * *p* < 0.05, ** *p* < 0.01, ns = not significant; **(A,I)** Kruskal-Wallis test with Dunn’s *post-hoc* test, **(B)** RM one-way ANOVA with Dunnett’s *post-hoc* test, **(C–G)** Student’s t-test, **(H)** one-way ANOVA with Tukey’s *post-hoc* test. Each dot in all assays represents an independent biological trial. Glycerol avoidance assays for the same strains are shown in Supplementary Figure S5. Additional data included in Supplementary Table S20.

Because we observed that loss of ADL function affects brood size (Figure 6D), we wondered whether loss of a functional germ line could result in defective ADL function. To test this hypothesis, we first performed ascr#3 avoidance assays using a *glp-1(q224)* mutant strain. The GLP-1/Notch receptor is necessary for the maintenance of the germline stem cell niche through promoting mitotic division of germ cells; thus, loss of GLP-1 causes the few present germline stem cells to prematurely enter meiosis and progress through spermatogenesis without germ stem cell renewal. At the restrictive temperature of 25°C, germline proliferation is prevented in the temperature-sensitive *glp-1(q224*) mutant (Austin and Kimble, 1987; Kimble and Crittenden, 2005). *Glp-1* expression was below our threshold of 10 RPKM in ADL neurons of control adults; thus, our experiment should be measuring the contribution of GLP-1 in the germline. As a control, we showed that ascr#3 avoidance by *osm-9(ky10)* adults was reduced compared to wild-type as previously shown (Figures 5C, 7A) (Jang et al., 2012; Sims et al., 2016). However, reversals by *glp-1* mutants at the restrictive (25°C, no germ lines) temperature were not significantly different from wild-type despite having slightly lower responses on average (Figure 7A).

A previous study found that a number of sperm genes, including *gsp-4*, were required for temperature tolerance and were mediated by the ASJ neurons (Sonoda et al., 2016). Since *glp-1(q224)* mutants produce a few sperm at the restrictive temperature, we next tested whether signals from sperm specifically are required for ADL function. We examined ascr#3 avoidance in strains carrying mutations in somatic sex determination genes, *fem-1* and *fog-2*, which result in hermaphrodite germline feminization and the production of only oocytes (Doniach and Hodgkin, 1984; Schedl and Kimble, 1988; Goodwin and Ellis, 2002; Ellis and Schedl, 2007). Reversals in response to ascr#3 by control female *fem-1(hc17)* and *fog-2(q71)* mutants were not statistically different from that of the wild-type strain. However, these strains exhibited the high variability in their avoidance behavior across trials, ranging from 10% to 80% and 22% to 80% of animals responding for *fem-1* and *fog-2* animals, respectively (Figure 7B). *fem-1* expression is above, and *fog-2* slightly below, our RPKM cutoff for ADL-expressed genes. In addition to promoting the production of sperm in hermaphrodites, noradrenergic signals required to maintain oocyte quiescence involve FEM-1 and FOG-2 functions (Kim et al., 2021); thus, we cannot rule out that their variable behavior is due to their function in ADL and not from the germ line. Collectively, our results are consistent with a model that ADL function contributes to hermaphrodite fecundity, but that germ cells are not required for ADL function.

### 3.8 Endogenous small non-coding RNA pathway genes mediate avoidance response to ascr#3

We have previously shown that, in addition to NRDE-3, the AGO ERGO-1 and components of the *Mutator* focus (MUT-16 and MUT-15) are critical for the regulation of *osm-9* expression and modulating ascr#3 avoidance behavior as a result of developmental history. Additionally, control adults harboring a mutation in genes involved in endogenous RNAi pathways, including *Mutator* focus components (*mut-14*, *mut-15*, and *mut-16*), *nrde-3*, and *ergo-1* exhibited statistically decreased ascr#3 avoidance when compared to wild-type (Sims et al., 2016). With the exception of *ergo-1*, these genes are lowly expressed in ADL neurons according to our RNA-seq data, beneath the 10 RPKM cutoff (Supplementary Table S2). We wondered whether lesions in additional endogenous small non-coding RNA pathways genes that are ADL-expressed would also affect ascr#3 avoidance behavior. First, HRDE-1/WAGO-9 is the germline-specific AGO counterpart of its NRDE-3 somatic paralog. HRDE-1 shares some of the same protein cofactors as NRDE-3 (NRDE-1, NRDE-2, and NRDE-4) to exert its heterochromatin formation and gene silencing roles (Guang et al., 2008; Burkhart et al., 2011; Ashe et al., 2012; Buckley et al., 2012; Shirayama et al., 2012; Billi et al., 2014). Unlike NRDE-3, HRDE-1 is critical for transgenerational epigenetic inheritance (TEI), a germline-dependent inheritance of a response to a trigger that persists for at least two to three generations beyond the original trigger (Ashe et al., 2012; Buckley et al., 2012; Gu et al., 2012; Shirayama et al., 2012; Rechavi and Lev, 2017; Perez and Lehner, 2019). We observed that *hrde-1* mutants have a slight, but not significant, increase in ascr#3 avoidance compared to wild-type (Figure 7C).

Next, we examined for a role in ascr#3 avoidance of some additional siRNA associated AGOs expressed in ADL neurons. WAGO-1/R06C7.1 and WAGO-4/F58G1.1 are two germline-specific proteins belonging to the WAGO clade (Yigit et al., 2006). WAGO-1 was shown to localize to P granules, the largest constituent of RNA-protein perinuclear condensates (or nuage) that accumulate in the cytoplasmic side of the nuclear envelop and are believed to be hubs where siRNA amplification and AGO distribution occur (Gu et al., 2009; Shirayama et al., 2012; Wang et al., 2014; Aoki et al., 2021; Sundby et al., 2021; Ouyang and Seydoux, 2022). WAGO-4 localizes to the Z granule component in nuage and is a necessary factor for RNAi inheritance (Wan et al., 2018). Comparison between wild-type adults and the *wago-1(ok1074)* mutant did not result in a significant difference in avoidance behavior to ascr#3 (Figure 7D). The *wago-4(tm1019)* mutants, however, showed statistically greater avoidance to ascr#3 compared to wild-type (Figure 7E). This suggests that, in addition to the requirement of *Mutator* proteins for ascr#3 avoidance (Sims et al., 2016), proteins known to localize in the germline Z granules may also play a role. Although ascr#3 exposure has no identified effects in future generations, exposure to ascr#3 during early larval development can affect how animals react to ascr#3 upon reaching adulthood (Hong et al., 2017). Notably, formation of germ granules in somatic tissue has not been detected to date, suggesting that certain AGOs may have different molecular functions in the soma compared to the germ line (Seroussi et al., 2023).

Next, we examined genes we found expressed in ADL neurons that function in different small non-coding RNA pathways, including the PIWI pathway. PRG-1 is a PIWI class AGO that associates with piRNAs (also known as 21U-RNAs) to safeguard the germ line by silencing detrimental mobile genetic elements. PRG-1 shares 91% amino acid identity with a second PIWI protein, PRG-2. PRG-1 and PRG-2 function in the germ line but unlike a *prg-1* mutant, mutation of *prg-2* does not result in aberrant accumulation of piRNAs (Batista et al., 2008; Das et al., 2008). Ascr#3 avoidance assays conducted on control adult *prg-1(tm872)* and *prg-2(tm1094)* mutants showed slight increases in avoidance behavior that were not significantly different compared to wild-type control animals (Figures 7F,G). Furthermore, PPW-1/WAGO-7 and PPW-2/WAGO-3 are members of the PIWI clade of AGOs (Yigit et al., 2006). PPW-1 is expressed in the germ line, intestine, and somatic gonad and is required for exogenous and endogenous RNAi (Tijsterman et al., 2002; Vasale et al., 2010). Most pseudogenes, long intergenic non-coding RNAs (lincRNAs), and transposable elements are targeted by a small cohort of AGOs that include PPW-1 (Seroussi et al., 2023). Expression of germline-specific PPW-2 has been detected in the spermatheca and in sperm where it associates with sperm-specific germ granules and paternal 22G RNAs to promote paternal epigenetic inheritance (Schreier et al., 2022; Seroussi et al., 2023). In addition to targeting spermatogenic genes, PPW-2 also targets repetitive elements and silences transposons (Vastenhouw et al., 2003; Seroussi et al., 2023). Avoidance to ascr#3 was not significantly altered in the *ppw-1(pk2505)* or *ppw-2(tm1120)* mutant strain, although *ppw-1* mutants exhibited slightly decreased avoidance compared to wild-type CON adults (Figure 7H).

Finally, we examined the role of paralogous ALG-1 and ALG-2 AGOs that associate with the miRNA class of small non-coding RNAs (Grishok et al., 2001; Yigit et al., 2006). *Alg-1* and *alg-2* mRNAs are expressed slightly below and above, respectively, our threshold cutoff for ADL expression. We measured the ascr#3 avoidance of each mutant strain given their relatedness with respect to their spatiotemporal expression patterns and overlapping function as miRNA-associated AGOs (Tops et al., 2006; Vasquez-Rifo et al., 2012; Aalto et al., 2018; Brosnan et al., 2021). ALG-1 and ALG-2 are ubiquitously expressed in somatic tissues with the latter also expressed in the germ line (Seroussi et al., 2023). *Alg-1(gk214)* mutant control adults exhibit a significant defect in ascr#3 avoidance indicating that ALG-1-associated miRNAs play a significant role in ADL function (Figure 7I). Although ALG-2 was found to be prevalently expressed in neurons over ALG-1 and is the chief direct interactor of pan-neuronal miRNAs (Maier et al., 2003; Redecker et al., 2003; Meizel, 2004; Ramírez-Reveco et al., 2017; Aalto et al., 2018; Brosnan et al., 2021), *alg-2(ok304)* adults showed no significant difference in ascr#3 response compared to wild-type (Figure 7I). Together, our results highlight the role that two major classes of endogenous small RNAs, namely, siRNAs and miRNAs, and their cognate AGOs play in ADL-dependent olfactory behavior.

### 3.9 The AGO pseudogene Y49F6A.1/*wago-11* is required for ADL neuronal function

**FIGURE 8:**
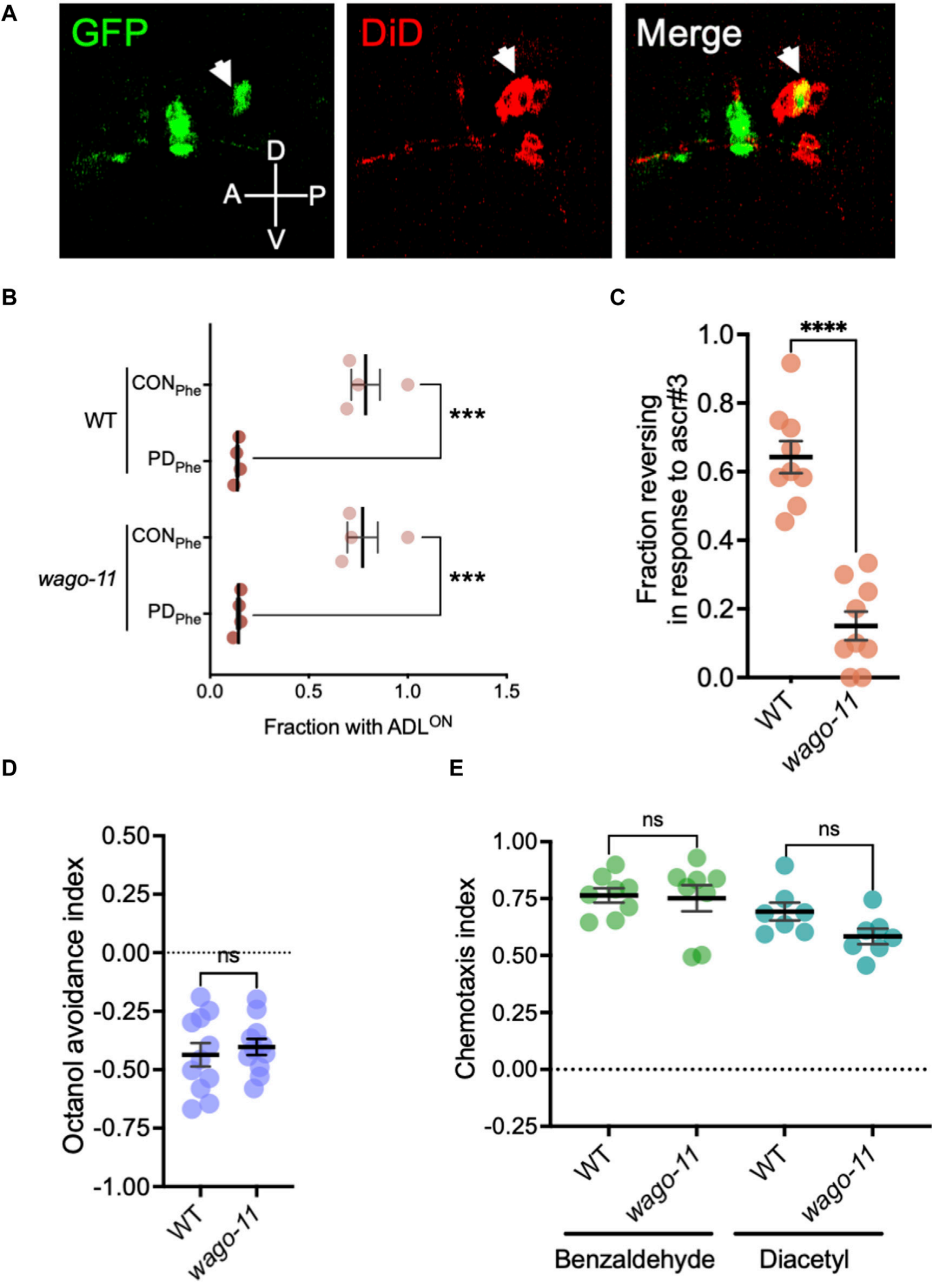
The AGO gene Y49F6A.1*/wago-11* is required for ascr#3 avoidance behavior. **(A)** Images of *wago-11*p*::gfp* fluorescent expression in the head of WT CON adult (green) stained with DiD fluorescence dye (red). DiD stains ADL and other amphid neurons in the head, including the ASK (anterior to ADL) and ASI (posterior to ADL) neurons that flank ADL. Merge image consists of overlapping the GFP fluorescent image with the DiD staining image. ADL is indicated by the white arrow heads. A, D, P, and V denote the anterior, dorsal, posterior, and ventral orientation of the animal. **(B)** Fraction of animals with ADL^ON^ status in wild-type and *wago-11* CON and PD_Phe_ 1-day adults expressing the *osm-9*p*::gfp* transgene reporter. **(C)** Fraction of 1-day old wild-type and *wago-11(tm1127)* CON adults that respond to ascr#3. **(D)** Avoidance index of 1-octanol by 1-day old WT and *wago-11(tm1127)* CON adults. **(E)** Benzaldehyde and diacetyl chemotaxis indexes of 1-day old WT and *wago-11(tm1127)* CON adults. **(B–E)** *** *p* < 0.001; **** *p* < 0.0001; ns = no significance (Student’s t-test). Each dot represents an independent biological replicate. Additional data included in Supplementary Table S20.

While examining endogenous RNAi pathway genes that were expressed in our ADL gene sets, we detected Y49F6A.1/*wago-11* expressed at extremely low levels in wild-type control and PD_Phe_ adults (average RPKM of <1 in each sample). Recently, WAGO-11 was designated as a pseudogene due its reported lack of expression from an endogenously-tagged *gfp* reporter, Western blotting, and low mRNA levels as detected with qRT-PCR (Seroussi et al., 2023). Given that *osm-9* (RPKM <7) and *nrde-3* (RPKM <5) function in ADL but also exhibit low ADL-expression levels (Sims et al., 2016), and the phylogenetic closeness between WAGO-11 and NRDE-3 (Yigit et al., 2006; Wedeles et al., 2013; Youngman and Claycomb, 2014), we sought to test if *wago-11* may also be functional in ADL neurons. First, we examined expression of an extrachromosomal transcriptional reporter consisting of 2 kilobases (kb) upstream of the *wago-11* start codon fused to *gfp* (*wago-11*p*::gfp*) injected into wild-type worms. We stained adults carrying the extrachromosomal array with DiD, a dye taken up by amphid neurons that allows for concise neuron identification. We found that *wago-11*p*::gfp* expression overlaps with ADL cell bodies and is present in additional unidentified head neurons (Figure 8A). Next, we examined whether WAGO-11 may play a role in the regulation of *osm-9*p::*gfp* expression by assessing the presence of GFP in control and postdauer adult ADL neurons. We found that *wago-11* mutants express the *osm-9* reporter like wild-type adults, indicating that WAGO-11 does not function in the developmental programming of *osm-9* in postdauer adults (Figure 8B). We then asked whether a *wago-11* mutant would exhibit a defect in ADL-dependent behaviors. Ascr#3 avoidance assays performed with *wago-11(tm1127)* mutant adults showed significantly lower reversal frequency compared to wild-type adults (Figure 8C), indicating that WAGO-11 is required for ascr#3 avoidance. Additional behavioral assays measuring octanol avoidance, benzaldehyde and diacetyl attractions showed that *wago-11* adults have similar responses to wild-type (Figures 8D,E). Thus, our results demonstrate that the WAGO-11 AGO is expressed in the ADL chemosensory neurons and plays a role in the regulation of ADL function.

## 4 Discussion

Neuronal and reproductive tissues are functionally intertwined to provide suitable and adaptive physiological responses to environmental changes. In *C. elegans* adults, transient passage through the dauer stage triggered by early-life stress results in the reprogramming of their transcriptome, chromatin states, and life history traits (Hall et al., 2010; Sims et al., 2016; Bharadwaj and Hall, 2017; Ow et al., 2018; Ow et al., 2021). Here we report that the adult postdauer transcriptome and the neuronal function of the ADL chemosensory neurons are reprogrammed following early-life crowding. We find that ADL neurons express germline-expressed genes, including those encoding MSPs, in a manner that is dependent on the somatic nuclear RNAi pathway AGO, NRDE-3. We also identify neuronal functions for the AGOs ALG-1, WAGO-4, and the previously uncharacterized WAGO-11, potentially expanding the roles of AGOs, primarily considered as custodians of germline integrity, in nervous system function (Supplementary Figure S6).

### 4.1 Gene expression regulation of MSPs in ADL neurons

The questions raised by our study on how and why sperm genes are expressed in neurons is a long-standing question in biology. In humans, the brain and testes share a striking similarity when comparing their transcriptome and proteome, with one study finding that a surprising ∼75% of sperm proteins are also expressed in neuronal cells (Matos et al., 2021). Consistent with this observation, mutations in genes expressed in neurons and sperm result in brain developmental defects and male infertility (Andersen et al., 2003). Some emphasis has been placed on understanding the role of neuronal genes in mammalian sperm function, particularly neuronal receptors and proteins that function in the calcium-dependent acrosome reaction for fertilization (Maier et al., 2003; Redecker et al., 2003; Meizel, 2004; Ramírez-Reveco et al., 2017). However, the function of sperm genes in neurons is less studied (Hu et al., 2016).

In multicellular organisms, cellular identity is determined by the set of genes expressed in that specific cell type. Related cell types derived from the same lineage often express transcription factors that drive gene expression, which defines that lineage (Bertrand et al., 2002; Nutt and Kee, 2007). Pan-neuronal gene expression, for example, is driven by DNA regulatory motifs in upstream regulatory regions that are bound by homeobox transcription factors (Leyva-Díaz and Hobert, 2002; Stefanakis et al., 2015; Reilly et al., 2020; 2022; Hobert, 2021). In *C. elegans*, *Drosophila*, and mice, identities of individual neurons are established and maintained via terminal selector transcription factors which, in conjunction with histone modification enzymes, promote expression of cell-specific neuropeptides, ion channels, and receptors and block expression of genes not part of the neuronal identity (Feng et al., 2020; Destain et al., 2024). Disruption of terminal selector genes results in a type of homeosis in the nervous system, where neurons change their identity to other types of neurons (Arlotta and Hobert, 2015).

In contrast, *C. elegans* hermaphrodite germline stem cells maintain totipotency to become either sperm or oocyte via mechanisms involving germline-specific perinuclear RNA granules (P granules), chromatin regulators, histone chaperones, and translation and transcription factors (Seydoux and Braun, 2006; Spickard et al., 2018; Fatima and Tursun, 2020; Marchal and Tursun, 2021). Germ cells that fail at totipotency are relegated to somatic fates with ensuing germline mortality and sterility (Updike et al., 2014). Oocytes that have disrupted P granules mis-appropriately express neuropeptide and sperm genes (Campbell and Updike, 2015; Rochester et al., 2022). The 3′ untranslated (UTR) regions of mRNAs are sufficient to regulate expression in most germ cells; however, sperm-expressed genes require 5′ UTRs to specify correct expression patterns (Shim, 1999; Merritt et al., 2008; del Castillo-Olivares et al., 2009; Kulkarni et al., 2012; Ragle et al., 2022). *Msp* gene expression requires a conserved GATAA motif in their 5’ UTRs that is bound by transcription factor, ELT-1, and the SET-17 H3K4me2 methyltransferase to maintain a permissive chromatin state at the *msp* gene cluster loci (Figure 3A) (del Castillo-Olivares et al., 2009; Engert et al., 2018).

In *C. elegans*, the 5′ UTR driven regulation of sperm genes is more similar to how neuron genes are regulated compared to other germline-expressed genes as described above. Our work here shows that MSP genes are not only expressed in wild-type control ADL neurons, but also that they are significantly differentially expressed in postdauer ADL neurons in a NRDE-3-dependent manner. We have shown previously that *osm-9* is downregulated in postdauer ADL neurons, which required NRDE-3 and the PD motif sequence in the 5′ UTR (Sims et al., 2016). Unlike *osm-9* expression, NRDE-3 is required for positive MSP expression in control adults instead of their downregulation in postdauer adults. Given the role of NRDE-3 in transcriptional silencing, these results suggest that NRDE-3 may be indirectly regulating MSPs (Supplementary Figure S6). Additionally, of the 21 *msp* genes differentially expressed in ADL, 19 genes were found to contain the ∼30 base pair conserved sequence element in the PD motif, which we have named the “MSP motif” (Figure 3A), further supporting our hypothesis that expression and regulation of MSPs in ADL is driven by upstream regulatory sequences (Sims et al., 2016). Our previous *osm-9* promoter analysis experiments demonstrated that the 5’ end of the MSP motif was required for *osm-9* expression in ADL neurons (Sims et al., 2016); thus, the MSP motif may also drive expression of the *msp* genes in ADL. Further experimentation will be necessary to determine the role of the MSP and E-box motifs in regulating differential expression of *msp* genes in ADL neurons (Figure 3A).

### 4.2 Potential MSP function in ADL

MSPs have two functions in *C. elegans* hermaphrodites to promote reproduction. First, they function in amoeboid-like sperm movement by forming filaments that promote lamellipod formation (Roberts and Stewart, 1995). MSPs were first discovered in *C. elegans* sperm (Klass and Hirsh, 1981), but much of our knowledge about the dynamics in sperm movement comes from studies in the *Acaris suum* nematodes. MSP dimers are added to growing filaments in the lamellipod at the leading edge of the cell to push to membrane forward (Italiano et al., 1996). MSP polymerization requires a membrane protein called MSP Polymerization Organizing Protein (MPOP) and cytosolic protein, MSP fiber protein 1 (MFP1); in contrast, MSP fiber protein 2 (MFP2) antagonizes this process to slow polymerization (Sepsenwol et al., 1989; Buttery et al., 2003; Grant et al., 2005). The filaments “treadmill” towards the trailing nucleus where MSP filaments are depolymerized, allowing the sperm to crawl over a substrate (Roberts and King, 1991). Mutant screens in *C. elegans* hermaphrodites have identified genes encoding proteins that play a role in this process, including the SPE-6 kinase required for filament assembly and the PP1 phosphatases, GSP-3 and GSP-4, for disassembly (Wu et al., 2012; Price et al., 2021). Our RNA-Seq data indicate that *C. elegans* genes encoding GSP-3 and GSP-4 are expressed in ADL neurons, while MFP1/MSD-1, MFP2/NSPH-2, and SPE-6 are not, suggesting that MSPs are not forming fibers in this context (Morrison et al., 2021). Our behavioral assays in this study demonstrated that the phosphatases, particularly GSP-3, are required for avoidance and attraction responses mediated by nociceptive and attractive sensory neurons, and rescue of GSP-3 in ADL is not sufficient to rescue the mutant phenotypes (Figures 5D,F,G). However, whether GSP-3 is expressed in other neurons and the targets of GSP-3 in ADL remain to be determined.

The second function of MSPs is to promote oocyte maturation and ovulation in the hermaphrodite gonad. MSPs are secreted from sperm and travel distally through the gonad to bind as an antagonist to the VAB-1 Ephrin receptor on the surface of arrested oocytes to promote their maturation. Additionally, MSPs bind to VAB-1 receptors in the gonad sheath cells to promote ovulation of oocytes through spermatheca for fertilization (Miller et al., 2003; 2001). VAB-1 is also expressed in the nervous system, and its role in neuronal morphogenesis, neuroblast movement, and axon migration has been well characterized (George et al., 1998; Zallen et al., 1999; Ghenea et al., 2005; Boulin et al., 2006; Mohamed and Chin-Sang, 2006; Mohamed et al., 2012). Whether MSPs expressed in ADL can be secreted and bind to neuronal VAB-1 receptors is unknown. Other proteins containing domains with similar structures to MSPs also have established roles in the nervous system. For example, a MSP-domain containing protein was identified in a screen for genes with roles in *C. elegans* axon guidance (Schmitz et al., 2007). In humans, a clinically relevant protein with a MSP domain is the vesicle-associated membrane protein-associated protein B (VAP-B) expressed in the nervous system, which plays a role in endoplasmic reticulum stress and the unfolded protein response among other cellular processes (Lev et al., 2008). The N-terminal MSP domain of VAP-B (MSPd) is cleaved and secreted from neurons and can bind as a ligand to Ephrin receptors (Tsuda et al., 2008). The P56S mutation in the VAP-B MSPd is associated with familial amyotrophic lateral sclerosis 8 (ALS8), and it alters the function of VAP-B by inhibiting cleavage of the MSPd and promoting formation of protein aggregates in neurons (Kanekura et al., 2006; Teuling et al., 2007; Kim et al., 2010). Additionally, the VAP protein in *Drosophila* localizes to neuronal cell bodies, and not in neuromuscular junctions, yet functions in regulating the latter (Tsuda et al., 2008). Interestingly, the cleaved VAP-B MSPd of humans and *Drosophila* can bind to the *C. elegans* VAP-1 Ephrin receptor and rescue oocyte maturity and ovulation defects in the gonad of sperm-deficient animals, suggesting evolutionary conservation of MSPd function in metazoans (Tsuda et al., 2008). VPR-1 is the *C. elegans* homolog of VAP-B, and it functions similarly by secreting its cleaved MSPd to signal through Ephrin receptors to regulate neuronal positioning and muscle function (Mohamed and Chin-Sang, 2006; Tsuda et al., 2008). Given the evolutionary conservation of function between the VAP-B and sperm MSP domains, we are testing the hypothesis that expression and potential secretion of sperm MSPs from ADL neurons affects VPR-1 function and Ephrin signaling.

### 4.3 Role of WAGO-11 in ADL neurons

Following its identification as an Argonaute (Yigit et al., 2006), the *wago-11* gene was categorized as a pseudogene because a *wago-11::3xflag::gfp* fusion protein was not visible and was poorly detected using protein blotting under normal growth conditions (Seroussi et al., 2023). Here, we provided evidence that *wago-11* is instead a functional gene expressed in ADL neurons with a critical role for ADL function. The discrepancies between our study and the previous work can be attributed to methodology differences as well as the nature of our experiments. First, we detected low *wago-11* expression using samples that were enriched for ADL cells rather than using whole animals, where *wago-11* mRNA would have been inevitably diluted. Second, to visualize *wago-11* expression, we used a multi-copy extra-chromosomal array instead of tagging the endogenous locus to express a *gfp* transcriptional reporter (2 kb upstream of the purported start site), which enabled its visualization using a standard epifluorescence microscope (Figure 8A) (Seroussi et al., 2023). Third, although our expression cut-off precluded the inclusion of *wago-11* in our analysis, we nevertheless proceeded to investigate its role due to its original Argonaute classification, and consequently identified it as a regulator of ADL function in adults (Figure 8C). The discovery of an uncharacterized Argonaute profoundly impacting ADL neuronal function and altering animal behavior is fertile ground for further questions, and our next step will be to decipher the role and mechanism of WAGO-11 function in ADL neurons.

## Data Availability

The original contributions presented in the study are included in the article/supplementary material. The data can also be found here: https://www.ncbi.nlm.nih.gov/geo/query/acc.cgi?acc=GSE268801.
