# Supplementary material for: RNAi-dependent expression of sperm genes in ADL chemosensory neurons is required for olfactory responses in Caenorhabditis elegans
Source: Front Mol Biosci. 2024 Jul 11;11:1396587. doi: 10.3389/fmolb.2024.1396587 (PMC11269235; doi:10.3389/fmolb.2024.1396587)
Supplement: Supplementary file 2 [file DataSheet1.docx]

Supplementary Material

# List of supplementary materials

Figure S1 (related to Figures 1A and 2A)

Figure S2 (related to Figure 4A)

Figure S3 (related to Figure 4C)

Figure S4 (related to Figure 4E)

Figure S5 (related to Figure 7)

Figure S6 (related to Figures 1-7)

# Table S1 Strains used in this study

# Table S2 Genes expressed in WT ADL CON and PD_Phe_ neurons

# Table S3 WT CON and PD_Phe_ genes in this study present in CeNGEN ADL transcriptome

# Table S4 WT CON and PD_Phe_ genes in this study present in pan-neuronal gene list

# Table S5 WT DE Up and Down genes in ADL

# Table S6 GO terms of WT Down genes with the most significant FDR statistical changes

# Table S7 GO terms of WT Up genes with the most significant FDR statistical changes

# Table S8 WT DE Down and Up genes in this study present in pan-neuronal gene list

# Table S9 WT DE Down and Up genes in this study present in germline-specific gene list

# Table S10 WT DE Down and Up genes that are spermatogenic- or oogenic-enriched

# Table S11 Genes expressed in *nrde-3* ADL CON and PD_Phe_ neurons

# Table S12 Comparison of WT ADL-expressed genes with *nrde-3* ADL expressed genes

# Table S13 GO terms of *nrde-3* Down genes with the most significant FDR statistical changes

# Table S14 GO terms of *nrde-3* Up genes with the most significant FDR statistical changes

# Table S15 *nrde-3* DE Up and Down genes

# Table S16 NRDE-3-dependent WT DE Up genes

# Table S17 NRDE-3-dependent WT DE Down genes

# Table S18 Genes with PD motif and conserved sequence motif in their 5’UTRs

# Table S19 Expression level of msp and msp-domain-containing genes

Table S20 Raw data corresponding to figures

# Supplementary Figures


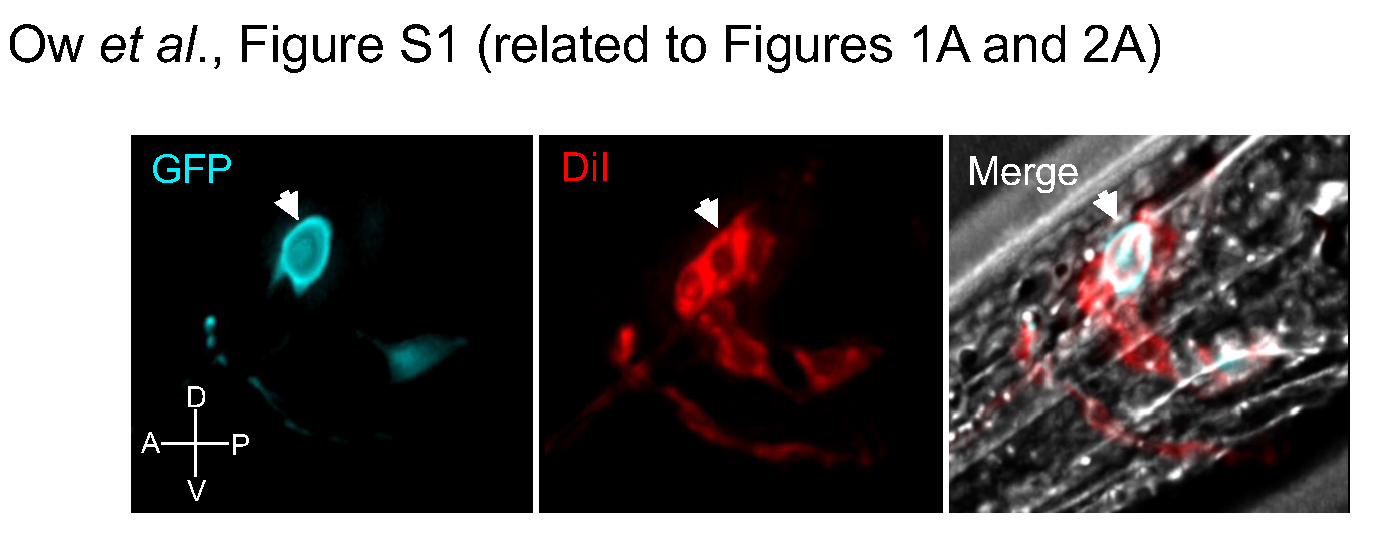


**Figure S1 (related to Figures 1A and 2A).** Images of wild-type, one-day old CON adult expressing the *sre-1*p*::gfp* (green) integrated transgenic reporter and stained with DiI fluorescence dye (red). Merge image consists of overlapping the GFP fluorescent, DiI, and DIC images. ADL is indicated by the white arrowhead. A, D, P, and V denote anterior, dorsal, posterior, and ventral orientation, respectively.

**
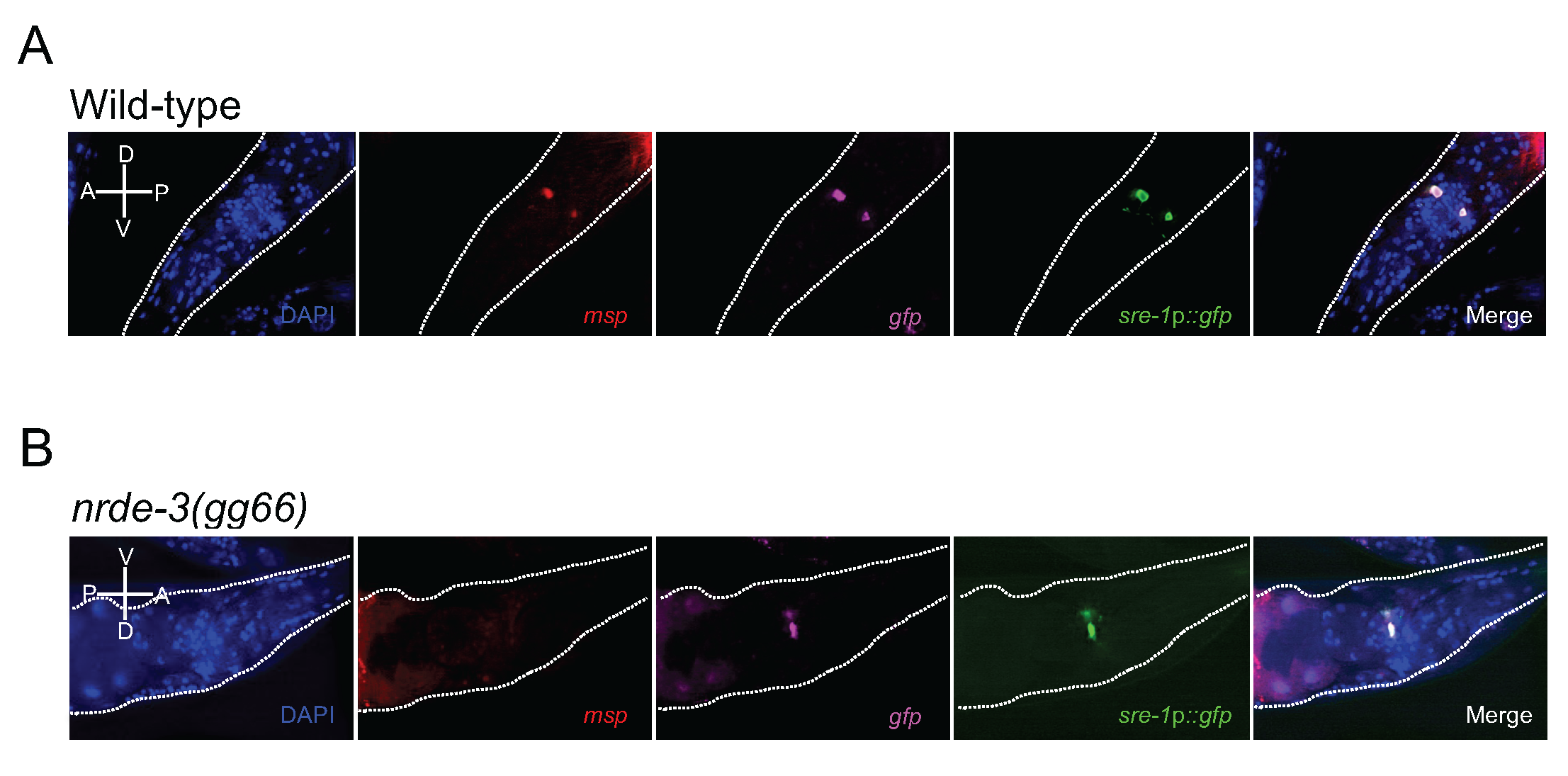
**

**Figure S2 (related to Figure 4A).** MSPs are expressed in ADL neurons. Representative images of smFISH of animal heads with probes against *msp* (red) and *gfp* (magenta) mRNAs using one-day old (A) wild-type and (B) *nrde-3(gg66)* CON adults. *sre-1*p*::gfp* transgene fluorescence (green) and DAPI stain (blue) also shown. Merge image consists of overlapping all the fluorescent images. The dashed line delineates the head of the animal. A, D, P, and V denote anterior, dorsal, posterior, and ventral orientation of the animal, respectively.

**
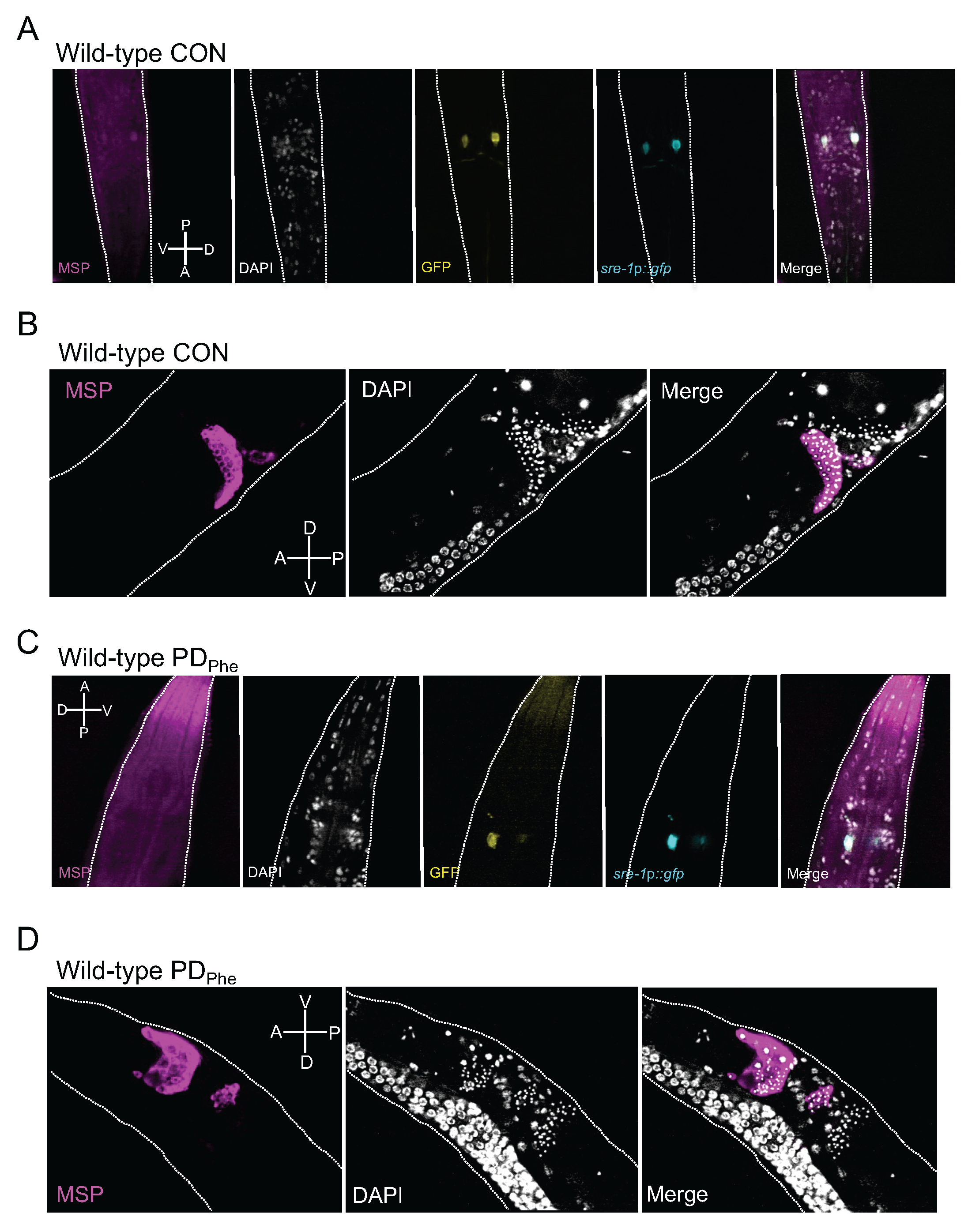
**

**Figure S3 (related to Figure 4C).** MSPs are expressed in ADL. (A, C) Representative immunofluorescence images of the (A) WT CON and (C) WT PD_Phe_ heads of the animals in Figure 4C using antibodies against MSP (magenta) and GFP (yellow). *sre-1*p*::gfp* transgene fluorescence (cyan) and DAPI stain (white) also shown. Merge image consists of overlapping all the fluorescent images. (B, D) Representative immunofluorescence images of the spermatheca using antibody against MSP (magenta) and DAPI stain (white) on one-day old (B) wild-type CON and (D) WT PD_Phe_ adults. For all images, the dashed line delineates the head or the gonad of the animal. A, D, P, and V denote anterior, dorsal, posterior, and ventral orientation of the animal, respectively.

**
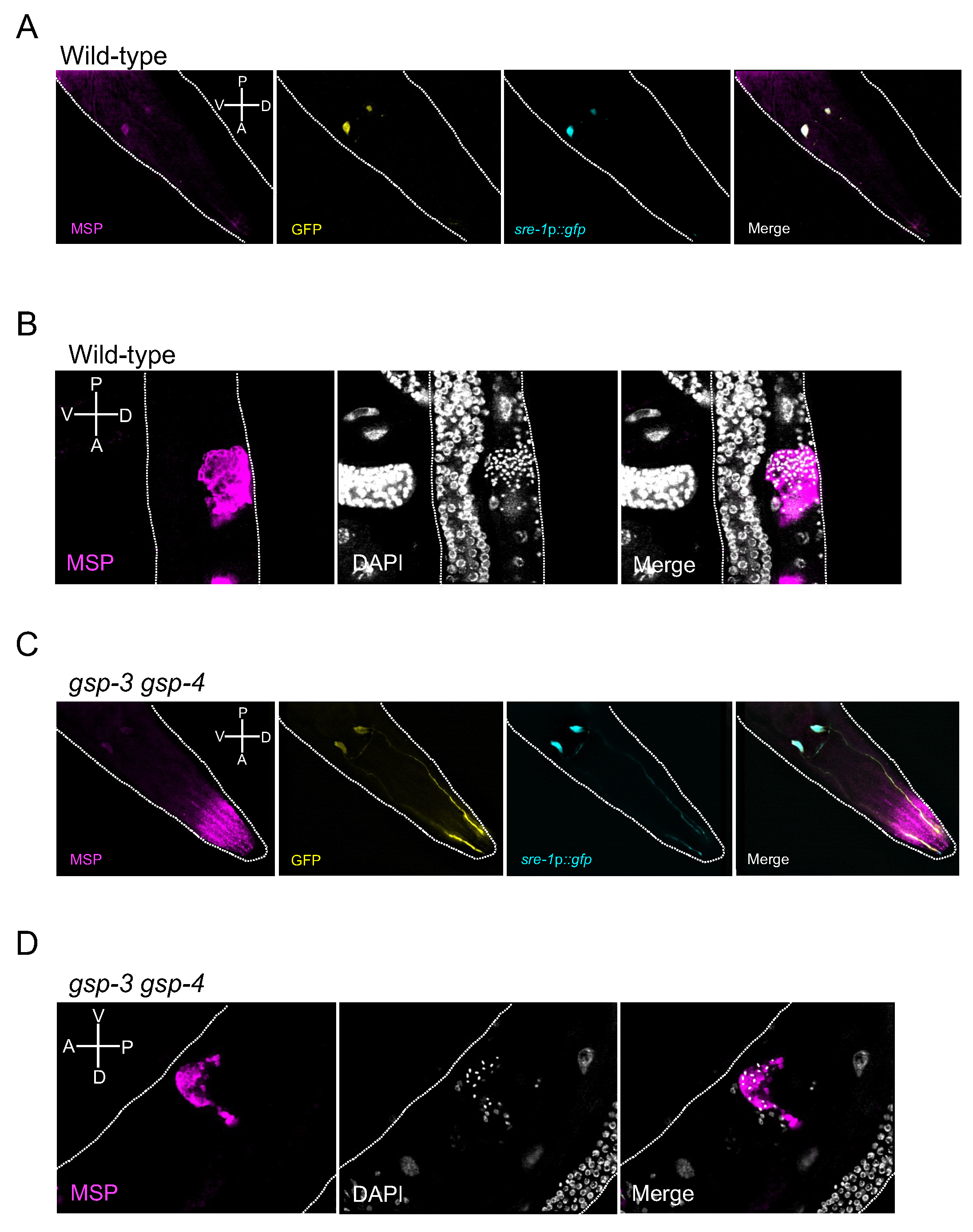
**

**Figure S4 (related to Figure 4E).** MSP level in ADL is decreased in *gsp-3 gsp-4* mutants. (A, C) Representative immunofluorescence images of the (A) WT CON and (C) *gsp-3 gsp-4* CON heads of the animals in Fig. 4E using antibodies against MSP (magenta) and GFP (yellow). *sre-1*p*::gfp* transgene fluorescence (cyan) and DAPI stain (white) also shown. Merge image consists of overlapping all the fluorescent images. Note that the contrast for the MSP image in (C) is increased to visualize the faint signal in ADL neurons. (B, D) Representative immunofluorescence images of the spermatheca using antibody against MSP (magenta) and DAPI stain (white) on one-day old (B) wild-type CON and (D) *gsp-3 gsp-4* CON adults. For all images, the dashed line delineates the head or the gonad of the animal. A, D, P, and V denote anterior, dorsal, posterior, and ventral orientation of the animal, respectively.

**
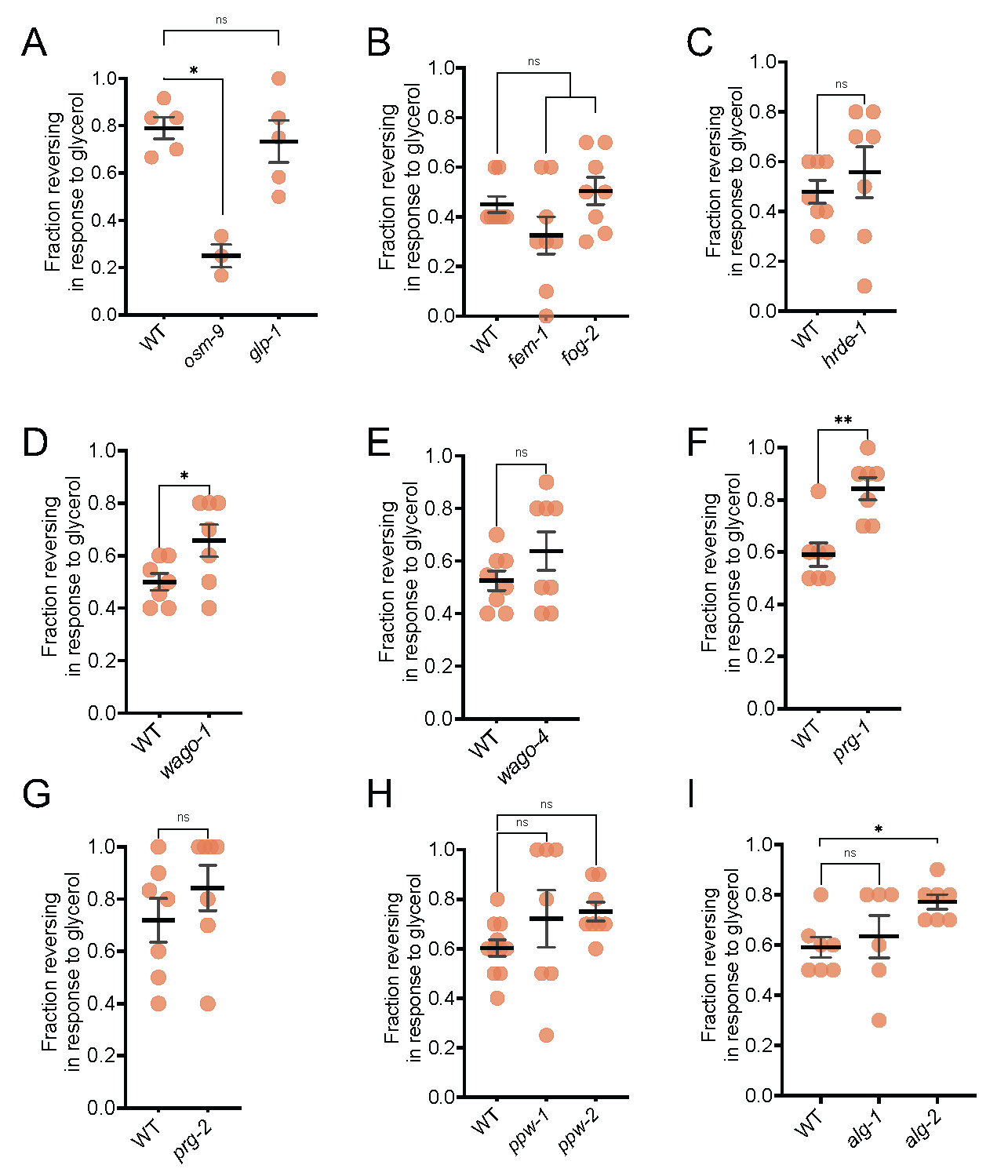
**

**Figure S5 (related to Figure 7).** Endogenous small RNAi pathways mediate glycerol avoidance response. (A-I) Fraction of one-day old CON adults reversing in response to glycerol for (A) wild-type, *osm-9(ky10)*, *glp-1(q224)*; (B) wild-type, *fem-1(hc17)*, *fog-2(q71)*; (C) wild-type*, hrde-1(tm1200)*; (D) wild-type*, wago-1(ok1074)*; (E) wild-type*, wago-4(tm1019)*; (F) wild-type, *prg-1(tm872)*; (G) wild-type N2*, prg-2(tm1094)*; (H) wild-type*, ppw-1(pk2505)*, *ppw-2(tm1120);* and (I) wild-type*, alg-1(gk214)*, *alg-2(ok304)* strains. * *p* < 0.05, ** *p* < 0.01, ns = not significant; (A, H, I) Kruskal-Wallis test with Dunn’s *posthoc* test, (B) one-way ANOVA with Tukey’s *posthoc* test, (C-G) Student’s *t*-test. Each dot in all assays represents an independent biological trial, also tested in Figure 7. Additional data included in Table S20.

**
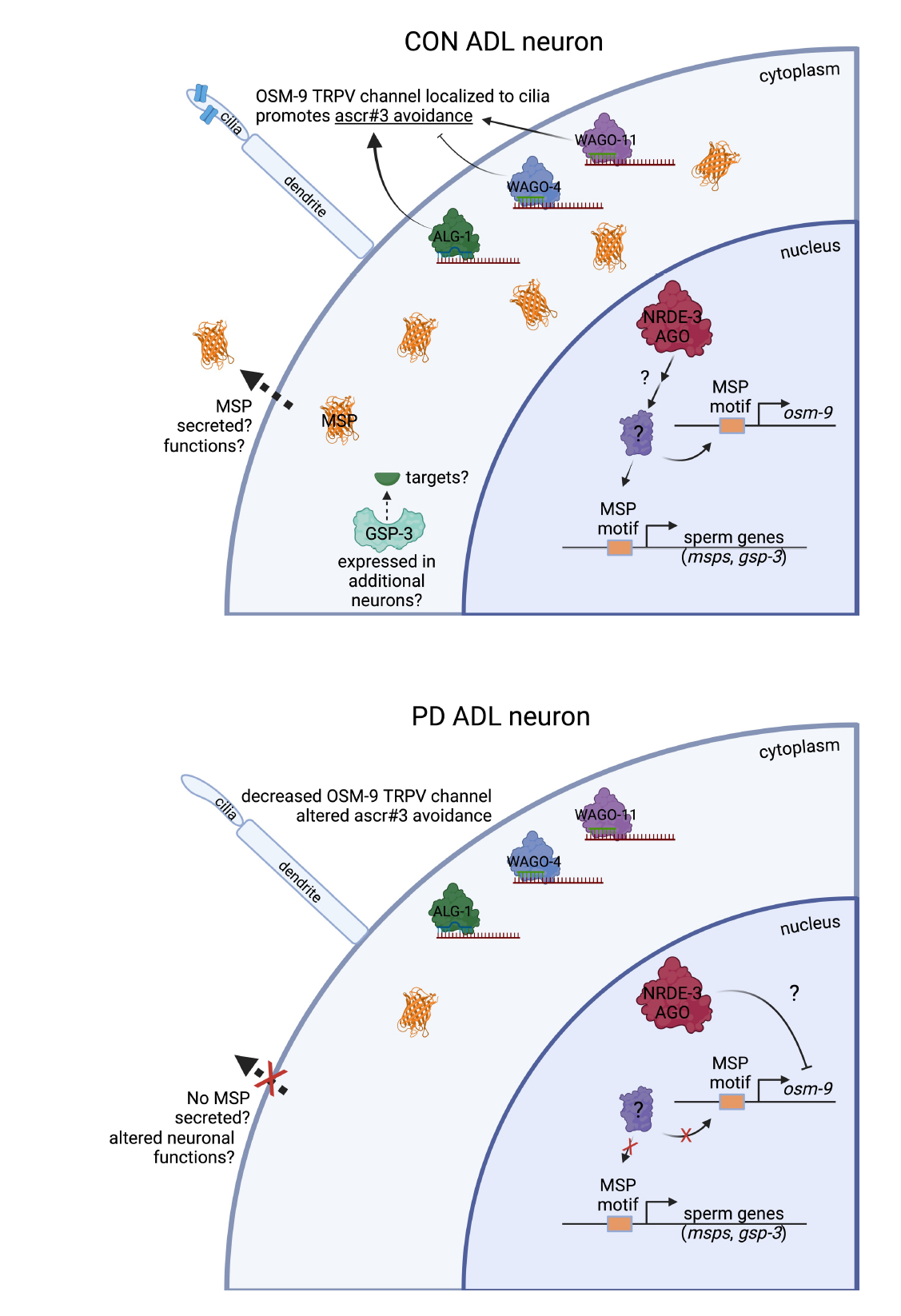
**

**Figure S6 (related to Figures 1-8).** Model for gene regulation in control and postdauer adult ADL neurons. Control adult ADL neurons express the *osm-9* TRPV channel gene and genes that are considered “sperm-expressed”, including *major sperm protein* genes (MSP) and the *gsp-3* protein phosphatase gene. NRDE-3 AGO is required for the expression of the sperm genes. Expression of additional Argonautes, ALG-1, WAGO-4, and WAGO-11, are required for the expression of *osm-9* as indicated. In postdauer adult ADL neurons, *osm-9* and sperm genes are downregulated. See manuscript for details.
